# Electrospinning Nonspinnable Sols to Ceramic Fibers and Springs

**DOI:** 10.1021/acsnano.3c12659

**Published:** 2024-05-08

**Authors:** Shiling Dong, Barbara M. Maciejewska, Ryan M. Schofield, Nicholas Hawkins, Clive R. Siviour, Nicole Grobert

**Affiliations:** † Department of Materials, University of Oxford, Parks Road, Oxford OX1 3PH, U.K.; ‡ Department of Engineering, University of Oxford, Parks Road, Oxford OX1 3PJ, U.K.

**Keywords:** coaxial electrospinning, sol−gel synthesis, ceramic nanofiber, helical structure, core−shell fiber

## Abstract

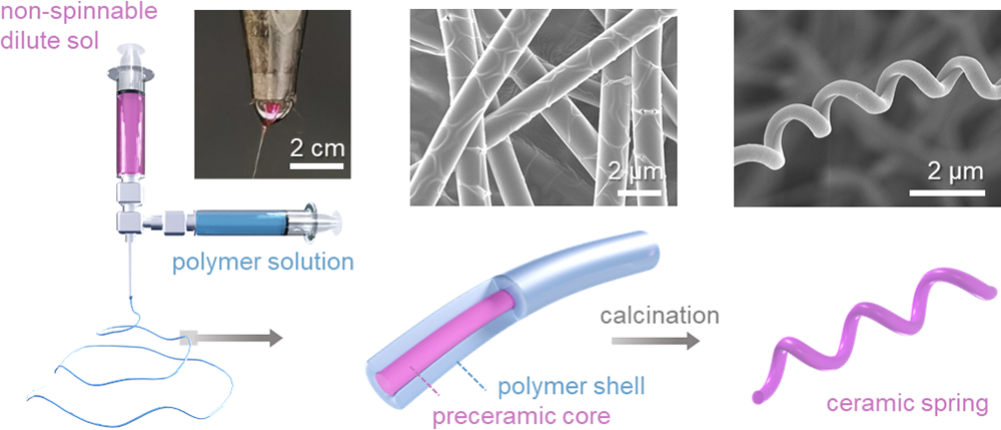

Electrospinning has been applied to produce ceramic fibers using sol gel-based spinning solutions consisting of ceramic precursors, a solvent, and a polymer to control the viscosity of the solution. However, the addition of polymers to the spinning solution makes the process more complex, increases the processing time, and results in porous mechanically weak ceramic fibers. Herein, we develop a coelectrospinning technique, where a nonspinnable sol (<10 mPa s) consisting of only the ceramic precursor(s) and solvent(s) is encapsulated inside a polymeric shell, forming core–shell precursor fibers that are further calcined into ceramic fibers with reduced porosity, decreased surface defects, uniform crystal packing, and controlled diameters. We demonstrate the versatility of this method by applying it to a series of nonspinnable sols and creating high-quality ceramic fibers containing TiO_2_, ZrO_2_, SiO_2_, and Al_2_O_3_. The polycrystalline TiO_2_ fibers possess excellent flexibility and a high Young’s modulus reaching 54.3 MPa, solving the extreme brittleness problem of the previously reported TiO_2_ fibers. The single-component ZrO_2_ fibers exhibit a Young’s modulus and toughness of 130.5 MPa and 11.9 KJ/m^3^, respectively, significantly superior to the counterparts prepared by conventional sol–gel electrospinning. We also report the creation of ceramic fibers in micro- and nanospring morphologies and examine the formation mechanisms using thermomechanical simulations. The fiber assemblies constructed by the helical fibers exhibit a density-normalized toughness of 3.5–5 times that of the straight fibers due to improved fracture strain. This work expands the selection of the electrospinning solution and enables the development of ceramic fibers with more attractive properties.

Electrospinning is the most robust and versatile technique for the mass production of high-quality nanofibers made of metals, ceramics, carbon, and composites.^1−3^ Among the broad selection of materials, ceramic fibers are particularly intriguing because their high-aspect-ratio geometry and large surface area endow them with properties and functions distinctive from their bulk counterparts, such as high chemical reactivity, excellent mechanical flexibility, elasticity, increased thermal and electrical conductivity, *etc*.^4−7^ So far, almost all electrospinning work to synthesize ceramic fibers starts with preparing viscous solutions that contain sol–gel reactants (such as alkoxides and metallic salts), a polymer carrier, a hydrolyzing agent (alcohol or water), an additive, and a solvent or solvent mixture.^8−10^ Such solutions are electrospun into precursor fibers, and the fibers are calcined at high temperatures to remove the organic components and trigger the crystallization of ceramics. This “sol–gel electrospinning” technique is currently the most popular way to generate ceramic fibers, where the role of a polymer includes (i) increasing the solution viscosity so that the polymer solution has sufficient extensional viscoelastic force to prevent the jet breaking into droplets,^11−13^ (ii) regulating the sol–gel transition to maintain the solution in a spinnable fluid state for a reasonably long time, i.e., several hours to days, and (iii) serving as a soft template to support the fiber morphology during the thermal conversion from a precursor to ceramic fibers.^4,14^

However, blending the polymer with the ceramic precursor solution results in many problems and limitations. The compatibility of the polymer with the solution systems is dependent on the solubility of the polymer and its interaction with the other components. Although many well-established precursor solution systems exist, modifying them by introducing other ions, tuning pH, or blending with other nanomaterial additives might trigger polymer participation, cross-linking, and phase segregation, making these solutions nonspinnable.^15^ Also, a good solvent is required to dissolve a certain amount of polymer to allow sufficient polymer chain entanglement.^13,16^ Yet, the ceramic precursors dispersed in such polymer/solvent medium tend to segregate into various phases due to their varied solubility, resulting in porous structures and unevenly distributed crystalline and amorphous phases.^17−19^ Possible phase separations in the solution also makes creating homogeneous multicomponent and high entropy ceramic fibers very challenging.^20−22^ Moreover, in the precursor fibers, the polymer intermixes with the sol–gel species and occupies a significant volume of the materials (typically about 40–60 wt %), removing the polymer leaves inorganic fibers with rough surfaces and pores. These structural defects severely deteriorate the mechanical properties of calcined ceramic fibers,^23,24^ limiting their installation in applications such as filtration, biomedical engineering, sensing, automobiles, energy generation, storage, *etc*.^25−28^

Only a few studies have reported electrospinning alkoxide sols without being blended in polymers. Choi et al. and Lee et al. produced SiO_2_ and TiO_2_/SiO_2_ fibers from aged alkoxide solutions,^9,23^ but the generated fibers have highly nonuniform diameters and beaded morphology due to the Rayleigh and electric field-induced axisymmetric instabilities.^29,30^ Huang et al. improved the spinnability of the SiO_2_/TiO_2_ solution by systematically evaluating the solution properties as functions of precursor composition, water content, and pH values, yet the obtained microfibers display unsatisfactory structural homogeneity.^31^ Recently, Ding’s group proposed a vacuum concentration process to remove the solvents partially and facilitate the precursors condensing into molecular chains and generating solutions with viscosities between 100 and 400 mPa s.^24,32,33^ This approach has enabled a series of ceramic fiber products with superior properties, although fundamentally, it still relies on tuning the rheological properties to create spinnable systems.

Coaxial electrospinning, a modified version of electrospinning, employs a spinneret consisting of two concentrically aligned nozzles. Two precursor solutions fed through the inner and outer nozzles are spatially separated, and the solidified fibers have a core–shell structure. Most studies on coaxial sol–gel electrospinning utilize polymer/sol blend solution as a shell to encapsulate oils, emulsions, and suspensions in the core,^34,35^ leading to hollow fibers,^36,37^ multichannel hollow fibers,^38^ tube-in-tube fibers,^39^*etc*. Nevertheless, these processes all require spinnable solutions with polymers blended in. Aziz et al. dissolved zinc neodecanoate in toluene and fed it through the core nozzle while flowing poly(vinyl alcohol) (PVA) solution through the outer nozzle to prevent jet breakage.^40^ ZnO fibers were obtained after removing PVA, but these fibers are nonuniform, porous, and interconnected into a web, indicating that the polymer and ceramic components are intermixed. To our understanding, applying a coaxial method to electrospin nonspinnable ceramic sols to create high-quality ceramic fibers has not yet been realized.

Herein, we propose a sol/polymer coelectrospinning technique, which uses a coaxial nozzle to encapsulate the dilute sol with a polymer shell solution, so that polymer-free sols with unlimited rheological properties can be electrospun. The resulting core–shell precursor fibers are then calcined into ceramic fibers in controllable diameters, which show reduced porosity, uniform and continuous fiber structure, and organized crystal packing compared to the conventional electrospun fibers. To demonstrate the universality of our strategy, a series of ceramic fibers made of TiO_2_, TiO_2_/SiO_2_, ZrO_2_, ZrO_2_/SiO_2_, and ZrO_2_/Al_2_O_3_ are prepared from nonspinnable sols. We also create ceramic micro- and nanosprings, examining the formation mechanisms by simulating the thermal and mechanical behavior of precursor fibers using a finite element method. Moreover, unlike the single-component polycrystalline TiO_2_ fibers previously known to be highly fragile, our TiO_2_ fibers exhibit exceptional flexibility and have a Young’s modulus of up to 54.3 MPa. The TiO_2_-based springs display a toughness 3.5–5 times higher than their straight fiber counterparts. We also present the single-component ZrO_2_ fiber, showcasing a Young’s modulus and toughness of up to 130.5 MPa and 11.9 KJ/m^3^, respectively, 5.4 and 2.5 times outperforming the ZrO_2_ fibers prepared using conventional sol–gel electrospinning techniques. This work could lead to more flexible, stronger, and tougher ceramic fiber materials with diverse geometries for a broad field of applications.

## Results/Discussion

### Sol/Polymer Coelectrospinning Technique for Nonspinnable Sols

**Figure 1:**
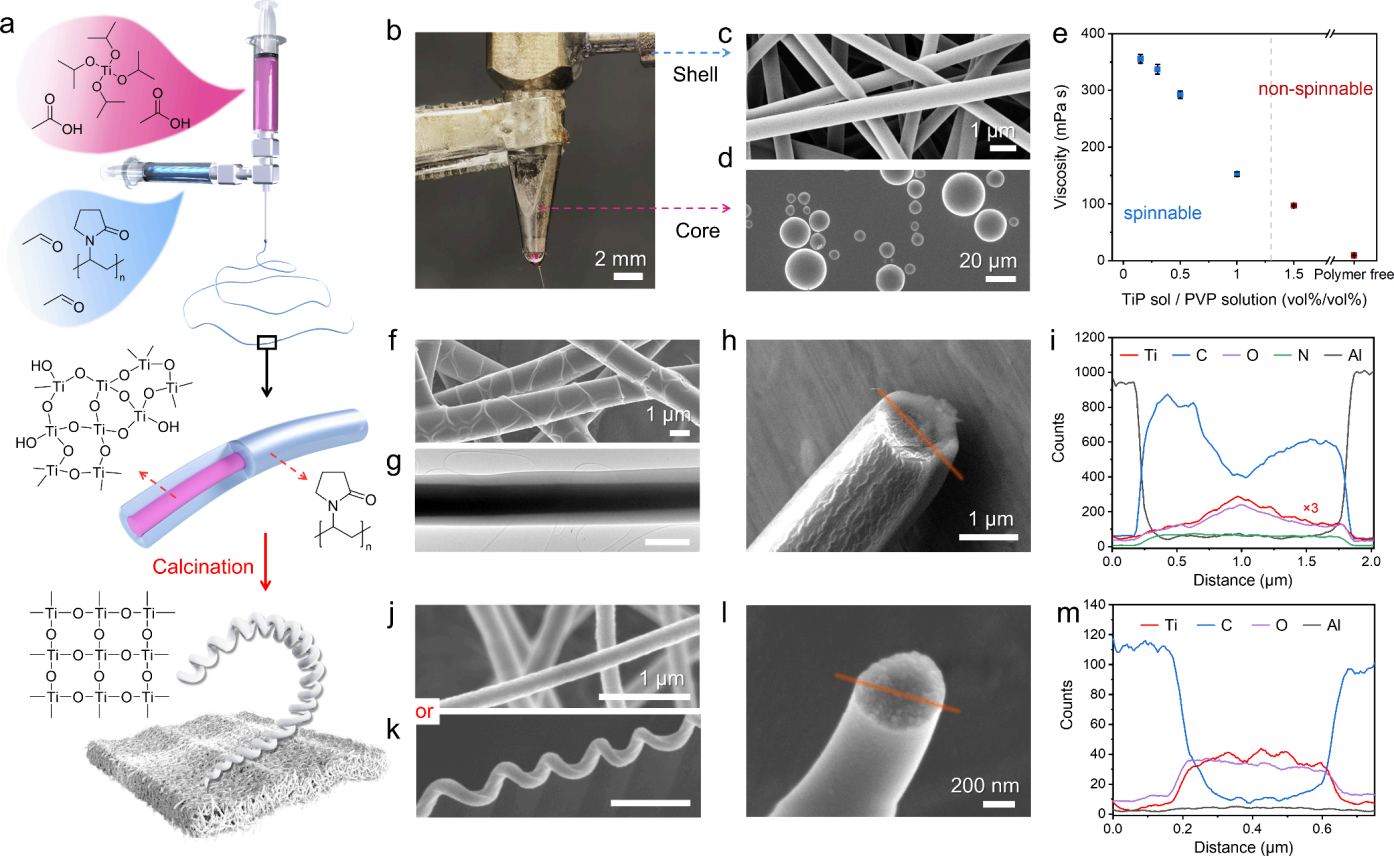
TiO_2_ fibers and springs electrospun from a nonspinnable dilute sol using the sol/polymer coelectrospinning technique. (a) Schematic illustration of feeding an alkoxide sol and polymeric solution through a coaxial nozzle. Electrospinning two solutions simultaneously generates precursor fibers with a core–shell structure. Calcining the as-spun fiber yields high-quality ceramic fibers, where the chemical composition of the sol–gel reactants and solid fibers is highlighted. (b) Digital photo of the bicomponent solution droplet at the tip of the coaxial nozzle, showing a clear boundary between the core and shell solutions. Scanning electron microscope (SEM) images of the electrospun product of (c) viscous polymer solution and (d) dilute alkoxide sol. (e) Plot of solution viscosity at varied alkoxide sol/polymer solution mixed ratios, where the spinnability is indicated. (f) SEM image shows the surface patterns of the as-spun precursor fiber, and (g) transmission electron microscope (TEM) images revealing its core–shell structure. (h, i) SEM and Energy dispersive x-ray spectroscopy (EDS) elemental line scan across the fiber cross-section suggests a core and shell layers, where the intensity of the Ti signal is magnified three times for better visualization. (j, k) SEM images of the calcined TiO_2_ fibers in straight nanofiber and coiled “nanospring” morphologies. (l, m) SEM and EDS elementary profile of the cross-section of a TiO_2_ ceramic fiber. The scale bar in f, g, j, and k is 1 μm.

The overall procedure of the proposed sol/polymer coelectrospinning technique is depicted in Figure 1a. TiO_2_ is presented as an example. A low-viscosity sol comprising only titanium isopropoxide (TiP) and acetic acid (AcOH) is fed through the inner core of a coaxial nozzle; simultaneously, a viscous polyvinylpyrrolidone (PVP)/ethanol (EtOH) solution is provided through the outer nozzle. Figure 1b and Figure S1 and Movie S1 in the Supporting Information display the bicomponent droplet at the nozzle tip. For better visualization, the core sol has been dyed a dark pink color using rhodamine B (4 mg/mL). Despite the miscibility between core and shell solutions and the high solubility of rhodamine B in EtOH,^41^ a sharp boundary between the core and shell part is consistently observed during coelectrospinning, indicating that the time scale of polymer/dye diffusion is much longer than the jet formation. Our results agree with the prior coaxial electrospinning studies that suggested that a miscible core–shell solution pair is essential for reducing the interfacial tension and allowing the shell solution to form a continuous sheath around the core.^42,43^ The substantially different rheological properties of core and sheath solutions can prevent solution mixing, resulting in compound fibers with distinct core–shell interfaces.^44^

When these two solutions are electrospun using a single nozzle setup, the dilute TiP sol (with a low viscosity of 9.2 mPa s) initially undergoes electrospraying and forms droplets on the collector surface, which then quickly is at the nozzle tip and ceases the process, therefore, nonspinnable (Figure 1d and Movie S2 (Supporting Information)). In contrast, the PVP solution with sufficient viscoelastic force can be electrospun into uniform submicron fibers on the substrate (Figure 1c). In conventional sol–gel electrospinning, the polymer solution is mixed with TiP sol. Figure 1e shows the range of mixing ratios when the resulting solution is viscous enough to be drawn into fibers by the electrostatic force. However, because TiP is highly reactive to moisture and condenses rapidly, TiP-based solutions cause frequent nozzle blockage, making the spinning process unstable and discontinuous.

In our proposed coelectrospinning method, the polymer solution serves as a shell enclosing the alkoxide sol which, on the one hand prevents the dilute liquid jet from breakage and on the other hand, decreases its solidification rate by reducing moisture exposure, allowing continuous and stable fiber production for hours. The as-obtained precursor fibers exhibit an average diameter of ca. 1 μm and a distinct surface pattern (Figure 1f). Such fiber consists of a 200–300 nm-thick PVP shell in an amorphous structure and a 400–500 nm preceramic core of aggregated TiO_2_ nanocrystals that is typically found in xerogels (Figure 1g and Figure S2 in the Supporting Information). These two parts display a sharp boundary in between. Energy dispersive x-ray spectroscopy (EDS) line scans across the fiber reveal additional Ti and O in the core and higher C and N contents in the shell (the Al signal comes from the Al foil used as the collector) (Figure 1h,i). To study the interface between the PVP shell and TiO_2_ core further, the as-spun fiber mats were soaked in water for days to remove the water-soluble PVP. The rinsed and dried fibers had an average diameter of 523 nm, matching the measured core size (Figure S3 in the Supporting Information). These fibers maintain an integrated network, which presents an FTIR spectrum with negligible absorption bands characteristic for PVP (e.g., C–N bond), attributed to the residue polymer between the fibers that requires breaking the integrated fiber network to remove. The washed fiber shows a rough surface due to the TiO_2_ condensates, yet there are no macropores between the condensates, suggesting the neglectable diffusion of polymer into the TiO_2_ core.^36^

After calcination, the obtained TiO_2_ fibers have a continuous structure and uniform diameter, proving that the polymer-free core forms a well-defined continuous core phase encapsulated by the PVP shell. Interestingly, the final morphology of the calcined fiber depends on the heat treatment route. When precursor fibers are attached to and supported by the substrate during calcination, the ceramic fibers remain straight (Figure 1j). If the fibers are peeled off and calcined in a free-standing mode, the ceramic fibers turn into ceramic micro- and nanosprings that have not been reported before (Figure 1k and Figure S4 in the Supporting Information). EDS line scans show uniform elemental distribution across the fiber cross-section, where the low C content is due to the thermal decomposition of the organic components (Figure 1l,m). The formation mechanisms of helical ceramic fibers made of various materials are discussed in the following sections.

Overall, our proposed sol/polymer coelectrospinning technique provides three advantages over standard sol–gel electrospinning: 1) this approach allows the nonspinnable dilute sols (e.g., viscosity <10 mPa s) to be electrospun directly, easing the efforts of blending polymers to tune the solution properties. There are a wide rage polymer shell materials which can be either miscible or immiscible with the sol.^35,45^ 2) The sol used herein is isopropoxide only diluted by AcOH, which undergoes neglectable changes in conductivity and viscosity with time because the ligand exchange reactions between the TiP and acetate groups barely trigger any nonhydrolytic sol–gel transition.^46^ Therefore, both core and shell solutions can be prepared as ‘stock solutions’ that remain stable over months. In comparison, once the precursor and polymeric solutions are mixed, the dynamic sol–gel transition gradually alters the solution properties, causing the solutions to be only spinnable for a short period, e.g., several days or hours.^47,48^ 3) Preparing the two solutions separately saves a lot of time as mixing them homogeneously is no longer necessary which could take weeks and is much more time-consuming than electrospinning itself.^49^

### Controllable Submicron and Nanoscale Fibers

**Figure 2:**
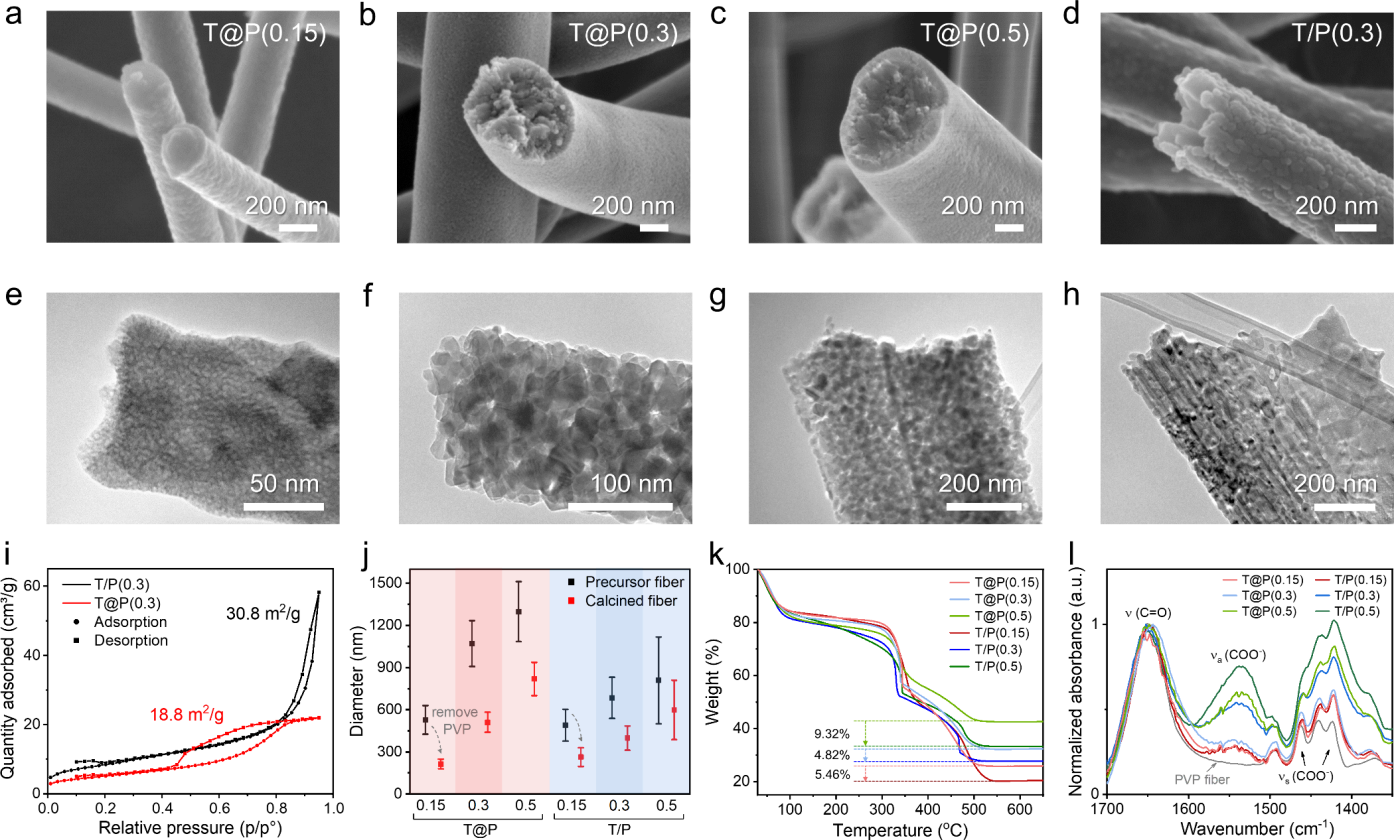
Characterization of TiO_2_ fibers obtained from coelectrospinning and single-nozzle electrospinning. SEM and TEM images of the core–shell TiO_2_@PVP fibers after calcination, i.e., TiO_2_ fibers (T@P), which are electrospun at sol feeding rates of (a, e) 0.15 mL/h, (b, f) 0.3 mL/h, and (c, g) 0.5 mL/h. (d, h) SEM and TEM images of the calcined TiO_2_/PVP hybrid fibers (T/P) from 0.3:1 ratio mixed precursor solutions and electrospun at 1.3 mL/h. (i) Nitrogen adsorption–desorption isotherms with calculated specific surface areas. (j) Summary of the fiber diameter distributions before and after calcination. (k) TGA analysis of the TiO_2_@PVP and TiO_2_/PVP precursor fibers, highlighting their different weight loss percentages. (l) ATR-FTIR spectra of six precursor fibers compared to the pure PVP fiber.

Our proposed sol/polymer coelectrospinning method yields core–shell TiO_2_@PVP fibers, which are further calcined into TiO_2_ fibers, named T@P. When the polymeric shell solution is fed at a constant rate of 1 mL/h, the average diameter of T@P increases from 213 to 819 nm upon increasing the core feeding rates from 0.15, 0.3, to 0.5 mL/h (Figure 2 a–c, Figure S5 in the Supporting Information). All of these fibers comprise TiO_2_ nanocrystals with even size and spherical shape, presenting a continuous structure and uniform morphology (Figure 2e–g). As the control group, TiP sol and PVP solution were mixed at volume ratios of 0.15:1, 0.3:1, and 0.5:1 and then electrospun using a single nozzle setup at feeding rates of 1.15, 1.3, and 1.5 mL/h, respectively. The as-spun hybrid TiO_2_/PVP fibers were subsequently calcined into T/P fibers. As shown in Figure S6 (Supporting Information), T/P(0.15) fibers display a flattened cross-section and are welded at the contact points because the precursor fibers with a low alkoxide content melt and collapse during calcination. T/P(0.3) and T/P(0.5) fibers are not continuous, with shortened lengths of typically <20 μm. This is because these fibers contain aggregated TiO_2_ crystals in nonuniform sizes and shapes (Figure 2d,h). Due to the weak bonds and irregular pores between the crystals, the fiber structure is brittle and weak, easily breaking into short pieces under thermal stress.

As shown in Figure 2i, T/P(0.3) displays a type IV isotherm with an H3 hysteresis loop and pore sizes mostly >20 nm.^50^ The coexistence of mesopores and macropores is due to the phase segregation induced by the polymer component, where the polymer-rich regions become more porous after calcination and polymer-lean regions contain ceramic nuclei that grow faster and undergo abnormal grain growth at the later calcination stage.^51^ In comparison, T@P(0.3) presents an H2 loop, which is typical for mesoporous materials. T@P(0.3) fiber has an average pore width of 6.1 nm and a narrower pore size distribution, corresponding to a total pore volume of about one-third of T/P(0.3) (Figure S7 in the Supporting Information). These mesopores originate from the interstices between the condensate particles in the TiO_2_ xerogel rather than due to phase separation, which can be further eliminated by optimizing the heat treatment.^52^ Moreover, T@P(0.3) displays a surface area of 18.8 m^2^/g, much smaller than the 30.8 m^2^/g of T/P(0.3), which is because TiP undergoes homogeneous polycondensation and crystallization without interaction with the polymeric component, leading to reduced surface defects.

The diameters of the precursor and TiO_2_ fibers produced from different solution recipes were evaluated statistically (Figure 2j). At the same TiP sol/PVP solution ratio, the core–shell T@P fibers exhibited larger diameters than the hybrid T/P fibers. The diameters of T@P decreased by 60–37% after calcination, i.e., more than the T/P fibers (46–26%, which indicates that T/P fibers have poorly compacted ceramic crystals and higher porosity of the fiber structure (Figure S8 in the Supporting Information). Thermal gravimetric analysis (TGA) was used to examine the thermal decomposition processes of precursor fibers produced by two electrospinning routes (Figure 2k). The desorption of moisture and removal of EtOH and AcOH residues occur at 25–120 °C. PVP side groups are released at 300–352 °C, and PVP carbon backbone cleaves at 352–500 °C.^53^ No weight loss was observed above 530 °C due to the complete decomposition of the organic components. Interestingly, the total weight losses of T@P precursor fibers was 5–9 wt % lower than those of T/P fibers with the same TiP/PVP ratio, suggesting that TiP consenses more readily in the core-shell fibers.

We employed attenuated total reflection-Fourier transform infrared spectroscopy (ATR-FTIR) to explore the interactions among TiO_2_, PVP, and acetate ligands in the precursor fibers. The penetration depth of infrared radiation can reach a few micrometers in polymers, therefore the ATR spectra contain information on the PVP shell and the TiO_2_ core of the fiber.^54^ The spectra in Figure 2l are normalized to the stretching vibration of the carbonyl group in PVP (ν(C=O)) at 1650 cm^–1^.^55^ The band at 1500–1600 cm^–1^ regions is the overlapping of several COO^–^ asymmetric stretching bands (ν_a_(COO^–^)) corresponding to different modes of TiP/AcOH complexation.^48^ The peaks at 1423 and 1463 cm^–1^ are assigned to the COO^–^ symmetric stretching (ν_s_(COO^–^)) of a Ti-acetate complex, where the carboxylate ligands can be coordinated in either bridging bidentate or chelating bidentate form (Figure S9 in the Supporting Information).^46^ The relative band intensities of ν(C=O) in T/P fibers are weaker than those in T@P, indicating the strong hydrogen bond between the surface hydroxyl groups of the TiO_2_ condensates and the C=O groups in PVP.^56^ The bonded PVP chains sterically block the reactive sites of TiP and hinder its hydrolysis, thereby preventing TiP from full condensation, in agreement with the TGA results.

Comparing three T@P samples, T@P(0.5) has a ν_a_(COO^–^) absorbance band with significantly higher intensity because the PVP chains coordinatively bond to the titanium acetate complexes and stabilize the acetate ligands. T@P(0.5) also has ν_s_(COO^–^) peaks that are more pronounced at 1423 cm^–1^ than at 1463 cm^–1^, suggesting that the Ti-acetate complex is dominated by a bridging bidentate configuration. Since only the bridging acetate group can coordinatively bond to PVP,^48^ these results indicate that T@P(0.5) has shell PVP solution partially mixed with the core TiP sol. In comparison, T@P(0.15) and T@P(0.3) have well-separated core–shell layers due to the thinner fibers and faster solidification, which minimizes interfacial mixing. The intensity of Ti–O–Ti stretching vibrations at about 800–600 cm^–1^ decreases with the reduction of the feeding rate of TiP sol, indicating the lower content of the preceramic TiO_2_.^57^

### Formation of Ceramic Micro- and Nanosprings

**Figure 3:**
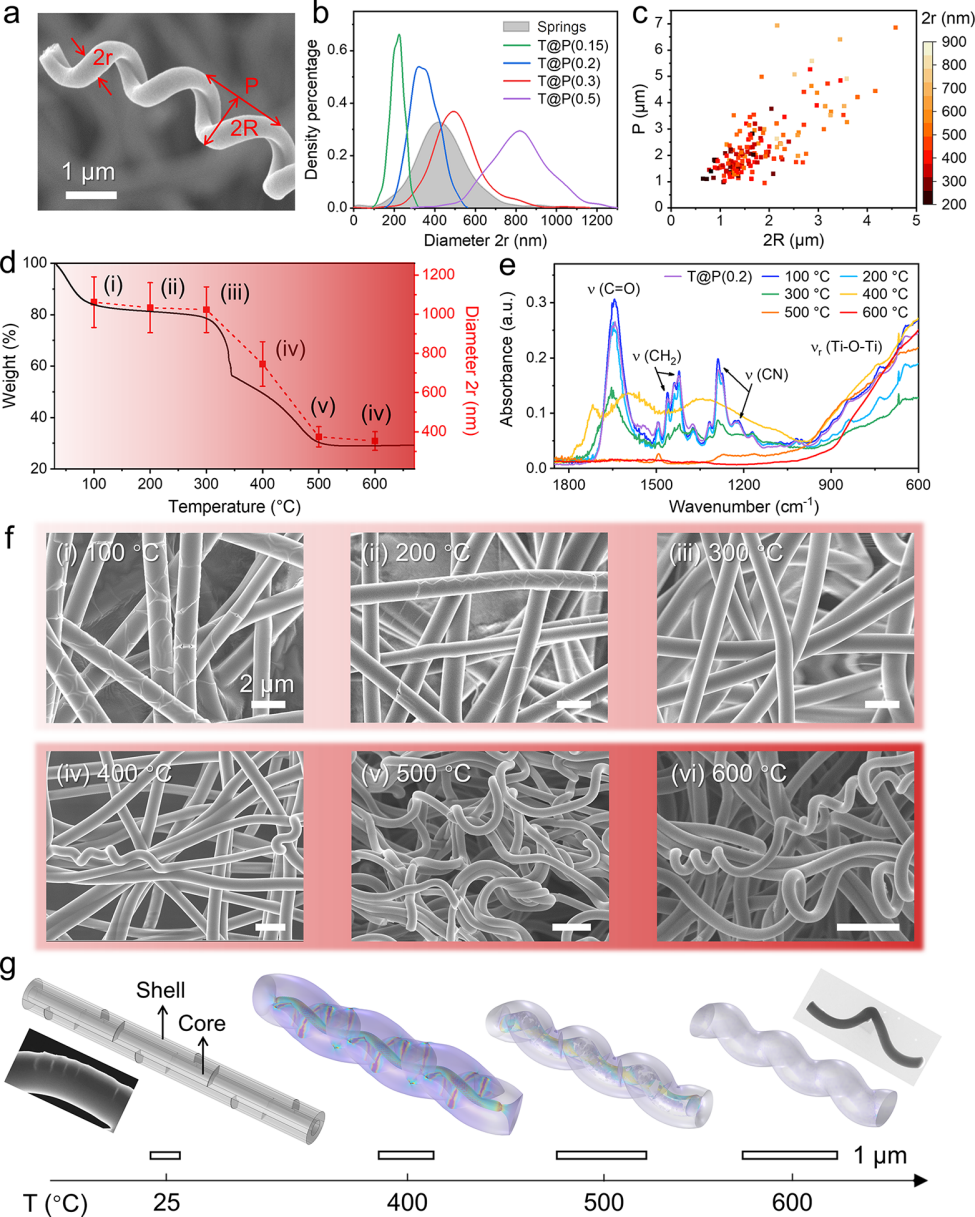
Formation of ceramic springs during thermal treatment. (a) SEM image of a TiO_2_ spring where the geometrical parameters, fiber diameter *r*, spring diameter *D*, and pitch length *P*, are labeled. (b) Population density percentage of the diameter of springs overlaid with the statistics of the diameter distribution of the T@P ceramic fibers produced at varied sol feeding rates. (c) Scatter plot of the 2*R*–*P* values of individual springs, where the color map indicates the fiber diameter (2*r*). (d) TGA curve of the core–shell precursor fiber that is produced using a 0.2 mL/h core solution feeding rate (T@P(0.2)), where the outer diameter of the fibers are labeled for each temperature. (e) ATR-FTIR spectra and (f) fiber morphologies examined every 100 °C from 100 to 600 °C. The scale bar in panel f (i)–(vi) is 2 μm. (g) COMSOL simulation of the thermomechanical deformation of 3D fiber models. The starting model has a core–shell structure, with “cuts” on the shell resembling the observed surface patterns in the scanning transmission electron microscope (STEM) image (inset). The thermal contraction and decomposition of the core and shell are modeled from 25 to 400, 500, and 600 °C, respectively. Scale bars depicted at each temperature are 1 μm. The color on the models represents the mechanical stress, and their scales are provided in Figure S15 (Supporting Information).

Helical structures in nature, such as tendrils,^58^ awns,^59^ and the xylem vessels of vascular plants,^60^ are formed due to asymmetric contraction of the cells and the chirality of the molecules. Electrospun fibers have potential in creating periodically curved structures because the electrified jets carrying multiple bending instabilities undergo mechanical buckling upon landing on the collector surface.^61,62^ However, helical fibers only form at the beginning of the electrospinning process, i.e., appearing at the first several layers of the deposited fiber, so prior work relied on generating helical fibers by blending different polymers and using non-centrosymmetric nozzles.^63−65^ Herein, we report the creation of ceramic springs based on the sol/polymer coelectrospinning method. Figure 3a shows a typical TiO_2_ spring, where the geometrical parameters are the fiber diameter (2*r*), spring diameter (2*D*), and pitch (*P*). We unbiasedly counted 200 ceramic springs observed in TiO_2_ fiber samples produced at different feeding rates. Figure 3b presents the population density of the diameter of the spring-shaped fibers (2*r*) (Figure 3b). Increasing the feeding rate of the TiP sol from 0.15 to 0.5 mL/h increases the fiber diameter, where T@P(0.2) and T@P(0.3) demonstrate the highest probability of forming spring structures. T@P(0.2) presents a fiber diameter distribution narrower than that of T@P(0.3), thus, it was selected for further study. Also, we observe that the pitch (*P*) and diameter (2*R*) of springs are positively correlated (Figure 3c). Fibers with larger diameters tend to form springs with larger *P* and 2*R*, probably because such TiO_2_ cores are stiffer and more resistant to bending into a higher curvature. When normalizing 2*R* and *P* with fiber diameter (2*r*), such a positive correlation relation remains, with the *R* and *P* mostly about 2–5 times and 5–16 times of *r*, respectively (Figure S10 in the Supporting Information).

To gain insights into the formation mechanism of the springs, the as-electrospun T@P(0.2) precursor fiber was heated in the air from room temperature to 600 °C, and the fiber diameter, morphology, and chemical bonds were monitored every 100 °C. The thermochemical conversion process can be divided into three stages (Figure 3d). The first stage covers 25 to 300 °C, where the fibers are approximately 1 μm in diameter, and the weight loss is mainly attributed to the removal of residual solvent and adsorbed water, showing no difference in the characteristic absorption bands (Figure 3e). A distinctive cleave-like pattern is observed at the surface of T@P(0.2) precursor fiber (Figure 3f (i),(ii)); such pattern remains significant even at 200 °C, above the PVP’s glass transition point of 150–180 °C, when the initial fiber shrinkage occurs due to the relaxation and realignment of the polymer chains.^66^ At 300 °C, PVP starts to decompose, suggested by the decreased intensity of the ν(C=O) band (Figure 3e,f (iii)). However, the thermal stress is preserved within the structure and influences further coiling deformation. The second stage, from 300 to 500 °C, is associated with the thermal decomposition of the PVP shell. The coiling of straight fibers is observed at 400 °C, when the average fiber diameter decreases to about 746 nm and the characteristic bands of PVP, e.g., ν(C=O) at 1645 cm^–1^, ν(CH_2_) at 1463 and 1423 cm^–1^, and C–N stretching at 1493, 1288, and 1217 cm^–1^, merge into broad bands (Figure 3f (iv)). The absence of PVP-related absorbance bands at 500 °C suggests that PVP is close to complete degradation, and a significant number of fibers display coiled helical structures (Figure 3f (v)). The third stage from 500 to 600 °C shows negligible weight loss, indicating that PVP is fully degraded. The fibers present a further decrease in diameter from 374 to 353 nm, accompanied by fibers evolving into micro- and nanosprings. In comparison, T/P(0.2) precursor fibers show similar progress of PVP decomposition as revealed by the FTIR spectra (Figure S11 in the Supporting Information). However, instead of coiling at any stage, T/P(0.2) fibers reduce in diameter at 400 °C, present a crystal structure at 500 °C, and undergo breakage when the polymer component fully decomposes at 600 °C (Figure S12 in the Supporting Information).

We propose a possible formation mechanism of fiber springs based on the thermomechanical deformation of core–shell precursor fibers. As shown in Figures 1f and 3f,g, the as-spun fibers have uniformly and periodically distributed cracks on the shell, separated by about 600–1000 nm. These cracks penetrate the fiber shell because they are permeation pathways for the encapsulated volatile solvent molecules to vaporize. However, the vapors produced by the core can be released through pinholes, not necessarily generating cracks that are primarily perpendicular to the fiber axis; therefore, we propose that the core–shell interaction during electrospinning also leads to crack formation. Specifically, at the initial stage of electrospinning, the viscous polymer solution dominates the shape and size of the sol jet because the dilute alkoxide sol has insignificant resistance to the electrostatic stretching. During jet thinning, the highly reactive sol rapidly condenses and solidifies into a rigid xerogel. This rigid core bonds to the shell at the interface and constrains the further thinning of the ductile polymer shell, causing the fiber surface to shrink against the stretching direction.^63,67^ Such longitudinal compression leads to cracks on the fiber shell, which distribute asymmetrically about the fiber axis and rotate along the change of the curving direction (Figure S13 in the Supporting Information). To validate these assumptions, we conducted coelectrospinning of TiP sol and PVP solution at a reduced polymer solution feeding rate of 0.5 mL/h. The resultant T@P(0.2/0.5) fibers have a thinner PVP shell that is less effective in blocking the vapor permeation and subjected to reduced constraints. No surface pattern is observed in these T@P(0.2/0.5) core–shell fibers, and no evident coiling or spiraling is shown after calcination (Figure S14 in the Supporting Information).

To evaluate the role of the cracks on the fiber shell in forming helical morphologies, we modeled the thermomechanical structural deformation of various patterned core–shell fibers. Detailed structural information and simulation parameters are given in Table S1 (Supporting Information). In short, fiber materials have mechanical modulus and coefficient of thermal expansion (CTE, negative values for thermal contraction) as a function of temperature. Anisotropic negative CTE was adopted for the polymer shell material, describing the polymer chain relaxation-induced volume shrinkage at low temperatures <200 °C^66^ and the thermal decomposition of polymer under a high-temperature oxidative atmosphere.^68^ Isotropic negative CTE was used for a TiO_2_ xerogel, simulating the shrinkage of the TiO_2_ xerogel core during calcination due to further condensation and crystallization.^69^ The parameters used for modeling were selected by quantifying the volume contraction of the fiber at different temperature ranges, but varying these parameters in reasonably wide ranges leads to similar results.

In the first stage, from 25 to 400 °C, we designed a simplified core–shell fiber with a 1020 nm shell, 480 nm core, and 10 μm length (Figure 3g, Figure S15 in the Supporting Information). The shell has multiple fan-shaped slits separated by 833 nm, and the slits are built consecutively by clockwise rotating the first cut by 90° about the fiber axis. The PVP shell is modeled as a soft polymer with longitudinal CTE one order smaller than the transverse CTEs because the thermal oxidation of polymer shells starts from the surface. The fiber core is a rigid material resembling a TiO_2_ xerogel, which contracts isotropically and more profoundly than the PVP shell at this temperature range. We hypothesize that the two materials with mismatched CTEs are bonded as a continuity, so the shrinking core imposes nonuniformly distributed stress on the cracked shell, which twists the straight fiber into a helix structure. After deformation, the fiber model has an average diameter of 760 nm, matching the observed fiber diameter at 400 °C. Note that the 90° fan-shaped slit shown here is an example; either narrowing or widening its angle can result in helix formation (Figure S16 in the Supporting Information). The second stage of calcination (from 400 to 500 °C) is associated with thermal decomposition of the PVP and densification of the TiO_2_ core. We used a more negative CTE for the PVP shell to simulate its degradation, and Young’s modulus was reduced by four orders of magnitude to model the softened state of the polymer before thermal breakdown. The deformed fiber model has a 370 nm diameter (controlled by the CTE values), in agreement with our observation by SEM. In the third stage from 500 to 600 °C, we modeled the helix fiber as a uniform polycrystalline material, which contracts isotopically into a ceramic spring with a final diameter of 350 nm.

Fiber models with varied asymmetric crack arrangements were also investigated. The core–shell model deforms into a straight spring when the cracks are distributed helically symmetrically about the fiber axis. If the relative angle of one pair of adjacent cracks changes from 90° to 180° and 270°, the spring bends at the middle point, but the handedness remains the same (Figure S17 in the Supporting Information). When the cracks are built with mirror symmetry about one fiber cross-section, the core–shell fiber develops into two helically twisted springs with different handedness; one observation is shown in Figure 3f (vi). These two springs are connected by a short straight section, similar to the perversion in the spiral plant tendril.^70^ When the cracks are distributed on one side of the fiber shell, the fiber curls during contraction, and the curvature depends on the span of the cracks on the fiber shell (Figure S18 in the Supporting Information). Furthermore, as mentioned, if the as-spun fibers are supported by a substrate during calcination, the straight-to-helical transformation becomes insignificant, probably because the substrate constrains the longitudinal shrinkage of the fiber mat. To verify that, we applied boundary load on the two ends of the fiber model in parallel to the fiber axis (Figure S19 in the Supporting Information). Upon increasing the tension stress, the deformed core–shell fibers have longer lengths, thinner sizes, and largely reduced helix radiuses. Note that although these fibers are under significant thermal and mechanical stress during deformation, their helical morphologies become stable after converting to ceramics. Therefore, these ceramic micro- and nanosprings are pioneering materials that combine the high rigidity of ceramic materials, the flexibility of nanocrystalline ceramics, and the potential elasticity of spring geometry.

### Mechanical Performance of Ceramic Fiber Assemblies by Sol/Polymer Coelectrospinning

**Figure 4:**
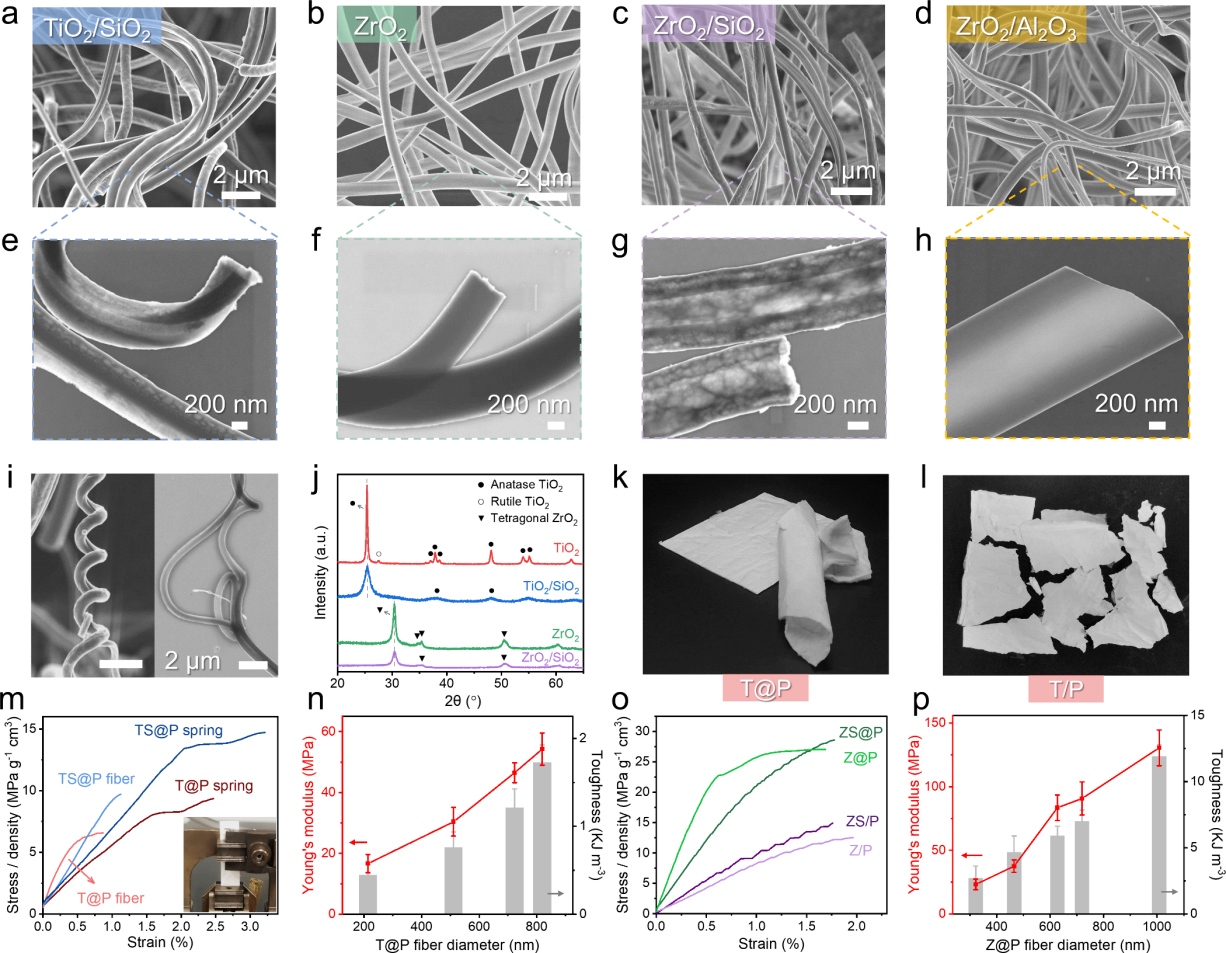
Production of various ceramic fibers and springs by the sol/polymer coelectrospinning technique. SEM and STEM images of (a, e) TiO_2_/SiO_2_ fiber (TS@P), (b, f) ZrO_2_ fiber (Z@P), (c, g) ZrO_2_/SiO_2_ fiber (ZS@P), and (d, h) ZrO_2_/Al_2_O_3_ fiber. (i) SEM and STEM images of TiO_2_/SiO_2_ ceramic springs. (j) XRD spectra of ceramic fibers obtained via co-electrospinning alkoxide sols. (k) Digital photo of the integrated and flexible T@P fiber mat compared to (l) the fragile T/P fiber mat broken into pieces. (m) Normalized tensile stress–strain curves of TiO_2_-based fibers in straight fiber morphology and spring shape. Inset: digital photo of a clamped fiber mat for the tensile test. (n) Young’s modulus and toughness of TiO_2_ fibers as functions of the fiber diameter. (o) Normalized tensile stress–strain curves of ZrO_2_-based fibers produced by coelectrospinning (Z@P and ZS@P) and conventional sol–gel electrospinning methods (Z/P and ZS/P). (p) Young’s modulus and toughness of ZrO_2_ fibers with varied diameters.

We applied our co-electrospinning technique to different sol/polymer systems, generating ceramic fibers composed of TiO_2_/SiO_2_ (TS@P), ZrO_2_ (Z@P), and ZrO_2_/SiO_2_ (ZS@P) (Figure 4a–c). Tetraethyl orthosilicate (TEOS) and zirconium isopropoxide (ZiP) were used as the precursors for SiO_2_ and ZrO_2,_ respectively. All prepared sols consist of alkoxides and solvents only, having low viscosities <10 mPa s, i.e., not spinnable by the conventional electrospinning approach (Figure S20 in the Supporting Information). These sols all exhibit Newtonian fluid properties under the tested range of shear rates due to the low polymerization degree of ceramic precursors.^32^ The coelectrospun precursor fibers show similar diameters because the viscous polymer solution shell dominates the elongation of the jet under the electric field. The fibers have distinctive microstructures because the alkoxide precursors have different reactivities and varied polycondensation reaction pathways. For example, TEOS is far less reactive than TiP and ZiP, which undergoes partial condensation during the solidification of the solution jet. The residue TEOS molecules evaporate with the solvents, resulting in a porous core of the precursor fibers (Figure S21 in the Supporting Information).^71^ After calcination at 600 °C, the SiO_2_-containing TS@P (Figure 4e) and ZS@P fibers (Figure 4g) are more porous than the single-component T@P (Figure 2b,f) and Z@P fibers (Figure 4f).

To prove the universal nature of our coelectrospinning method, we present the ZrO_2_/Al_2_O_3_ fiber synthesized from dilute Zr/Al sol (Figure 4d,h and Figures S21 and S22 in the Supporting Information). Differing from previous solutions in which highly reactive TiP and ZiP are dispersed in a nonaqueous medium, in this case, a mixture of Zr/Al salts and alkoxides are dissolved in H_2_O. This metal salt-based Zr/Al sol has a low viscosity of *c.a.* 5 mPa, which is then coelectrospun with the PVP/EtOH shell solution and calcined to ZrO_2_/Al_2_O_3_ fibers. This ZrO_2_/Al_2_O_3_ fiber, however, is not further investigated in this work because its precursors are substantially different from the other four types of ceramic fibers. Yet, we prove that our proposed coelectrospinning method is compatible with both nonhydrolytic and hydrolytic sol–gel synthesis routes, which means that a boundless selection of sol–gel reactants can be used for precise composition control of the ceramic fibers.

By co-electrospinning TiP/TEOS sol, we created TiO_2_/SiO_2_ ceramic springs (Figure 4i and Figure S23 in the Supporting Information). The coiling behavior is similar to the formation mechanisms of the TiO_2_ springs, ascribed to the asymmetrical cracks on the polymer shell that induce torsion to the ceramic core during calcination. Interestingly, no helical ZrO_2_ and ZrO_2_/SiO_2_ fibers could be observed, even though the precursor fiber of ZrO_2_ displays surface patterns identical to those of TiO_2_ and TiO_2_/SiO_2_ precursor fibers. Reason for this could be that the thermal contraction of the solid ZrO_2_ core is insufficient to drive the helix formation and the ZrO_2_ core is too rigid to be deformed by the thermomechanical stresses. On the other side, ZrO_2_/SiO_2_ precursor fibers display no crack on the shell, most likely due to the lower solidification rate of ZiP/TEOS sol with a high concentrations of TEOS , which means the core and shell have more time time to dry.

Electrospun nanofibers made of single-component polycrystalline ceramics, such as TiO_2_ and ZrO_2_, are known to be highly brittle and fragile; thus, their assembled fiber mats are almost unusable for any practical applications.^72−75^ Improving the mechanical properties of these fibers requires the addition of high concentrations of dopants (e.g., silica and carbon) as well as the incorporation of a polymer to reinforce the fiber structure.^74,76−79^ However, adding these inactive binding materials can deteriorate the physical properties and chemical reactivity of crystalline ceramics. Herein, our sol/polymer co-electrospinning method creates mechanically robust single-component TiO_2_ and ZrO_2_ ceramic fibers as well as their composites with SiO_2_. X-ray diffraction (XRD) patterns reveal that the T@P fiber consists of TiO_2_ crystallites in mixed anatase and rutile phases, and the Z@P fiber contains a tetragonal ZrO_2_ phase (Figure 4j). The SiO_2_-composited fibers have lower crystallinity and smaller crystallite sizes because the amorphous SiO_2_ effectively inhibits the crystallization of TiO_2_ and ZrO_2_ (Figure S24 in the Supporting Information). Even though T@P fibers have a relatively large crystallite size of 37.4 nm, their assembly is highly deformable and flexible and can recover from bending and folding (Figure 4k and Movie S3 (Supporting Information)). In sharp contrast, the T/P fibrous mat obtained from conventional sol–gel electrospinning undergoes brittle fracture upon small deformation (Figure 4l and Movie S4 (Supporting Information)), which is ascribed to the discontinuous fiber structure and less organized crystal packing.

To evaluate the mechanical properties of the ceramic fibers, we employed a dynamic mechanical analyzer (DMA) equipped with a film tensile clamp (Figure 4m, inset). For each test, the ceramic fibrous mat was cut into a ca. 8 mm wide and 2 cm long strip. We compare their mechanical performance after normalizing the tensile stress–strain relations with the density of the fiber mat because the fiber morphology, porosity, and packing density vary with the electrospun precursor sol. Multiple as-measured stress–strain response results are also provided in Figure S25 (Supporting Information). Figure 4m compares T@P and TS@P fibers in two different morphologies. The straight TiO_2_ fibers present a normalized Young’s modulus nearly three times the springs, while TiO_2_ springs exhibit a fracture strain 2.8 times larger than the straight fibers. Similarly, TS@P springs have a toughness five times higher than the TS@P straight fibers due to the 3.2 times larger fracture strain. Note that T/P fibers cannot be tested by the DMA setup because the fiber materials are severely damaged upon mounting and fixing to the tensile clamps (similar observations were reported in refs (72, 74, and 79)). Figure 4n compares TiO_2_ fibers with diameters varying from nanoscale to submicrometer, controlled by the core flow rate. Without density-normalization, the Young’s modulus and toughness of TiO_2_ fiber increase with fiber diameters, reaching the maximal values of 54.3 MPa and 1.73 KJ/m^3^ when the average fiber diameter is 819 nm.

Figure 4o displays the normalized tensile stress–strain curves of ZrO_2_-based fibers, where ZrO_2_ and ZrO_2_/TiO_2_ fibers produced by conventional sol–gel electrospinning at the same solution feeding rates are denoted Z/P and ZS/P, respectively. The Z@P fibrous mat exhibits normalized Young’s modulus and tensile toughness that are 5.4 and 2.5 times those of Z/P. The ZS@P fibrous mat is 2.3 times stronger and 2.1 times tougher than ZS/P. The Young’s modulus and toughness of ZrO_2_ fiber mats also increase with the fiber diameter. A fibrous mat of ZrO_2_ microfiber with about 1 μm diameter presents a Young’s modulus of 130.5 MPa and toughness of 11.9 KJ/m^3^, which are nearly doubling those of ZrO_2_ submicron fiber with 600–750 nm diameter and almost four times those of thinner ZrO_2_ fiber with an average diameter of 321 nm. Overall, our proposed coelectrospinning technique allows the fabrication of various ceramic fibers with controlled sizes, leading to more mechanically robust fibers to meet the requirements for real-life applications.

## Conclusions

In this study, we employed a coaxial nozzle to electrospin dilute polymer-free sols enveloped within a polymer shell, enabling nonspinnable sols to form stable core–shell precursor fibers. The as-spun fibers are calcined to yield uniform and homogeneous ceramic fibers with diameters ranging from submicrometer to nanoscale. We also present a pioneering ceramic micro- and nanosprings, elucidating the helical formation by simulating the thermomechanical behaviors of core–shell fibers with patterned shell structures. To highlight the universal potential of our methodology, we reported five types of ceramic fibers from dilute polymer-free sols. Single-component polycrystalline TiO_2_ fibers with reduced porosity, fewer surface defects, and uniform structures were presented, which exhibit an excellent flexibility and Young’s modulus up to 54.3 MPa. TiO_2_ springs present a larger fracture strain than the straight fibers, showing a toughness that is 3.5 times higher. Additionally, we create single-component ZrO_2_ fibers with a Young’s modulus and toughness of 130.5 MPa and 11.9 KJ/m^3^, respectively, which are significantly superior to counterparts made through conventional sol–gel electrospinning. This research introduces a universal electrospinning approach for nonspinning solutions, which is envisaged to lead to a great variety of fiber materials with improved mechanical properties for diverse advanced applications.

## Methods/Experimental Section

### Materials

Polyvinylpyrrolidone (PVP, M.W. 1,300,000), titanium(IV) isopropoxide (TiP, 97+%), tetraethoxysilane (TEOS, 99+%), and aluminum chloride (AlCl_3_, anhydrous, granular) were purchased from Alfa Aesar. Zirconium(IV) propoxide (ZiP, ca. 70%, solution in 1-propanol) was purchased from Fisher Scientific. Acetic acid (AcOH) was purchased from Honeywell. Zirconium(IV) acetate hydroxide (ZrAc), aluminum nitrate nonahydrate (AN), aluminum isopropoxide (AiP), and ethanol (≥99.8% GC) were purchased from Sigma-Aldrich. All reagents were used without further refinement.

### Preparation of Alkoxide Sols and Polymer Solutions

For synthesizing TiO_2_ fibers, the dilute sol (core) was prepared by mixing 4.3 g of TiP and 1 g of AcOH at room temperature for 1 h. The polymer solution (shell) was prepared separately by dissolving 1 g of PVP powder in 10 mL of EtOH and stirring at 50 °C for 1 h. The polymeric solution for single-needle electrospinning was obtained by mixing these two solutions at different weight ratios, stirring at 65 °C for at least 3 h to get a clear yellowish solution. Detailed solution compositions are listed in Table S2 (Supporting Information).

For the generation of other ceramic fibers, the same polymer shell solution was used. The dilute sol was a mixture of (i) 3.5 g of TiP, 1.5 g of TEOS, and 1 g of AcOH for TiO_2_/SiO_2_ fiber, (ii) 2.75 g of ZiP and 1 g of AcOH for ZrO_2_ fiber, (iii) 1.6 g of TEOS, 2.4 g of ZiP, and 1 g of AcOH for ZrO_2_/SiO_2_ fiber, and (iv) 0.44 g of AlCl_3_, 0.7 g of AN, 0.7 g of AiP, and 3 g of ZrAc dissolved in a mixture of 4 mL of H_2_O, 3 mL of EtOH, and 1 mL of AcOH for ZrO_2_/Al_2_O_3_ fiber. Solutions (i)–(iii) were mixed at room temperature for 1 h. Solution (iv) was stirred at 80 °C for 3 h.

### Electrospinning

Sol/polymer coelectrospinning was performed using a coaxial nozzle (22G inner needle and 14G outer needle) attached to a high voltage supply (Genvolt High Voltage Power Supply). Single nozzle electrospinning was performed using an 18G needle. A Fusion 4000-X Dual Motor Pump (KR Analytical Limited) was used to feed the solutions at the designated rates. The detailed solution feeding rates for TiO_2_ fibers are given in Table S2 (Supporting Information). The diameters of the ZrO_2_ fibers were controlled in a similar manner. The fibers were collected on a piece of Al foil taped on a grounded metal substrate. The nozzle-to-substrate distance was kept at 25 cm. After electrospinning, the precursor fibers were either directly placed (attached to Al foil) or peeled off from the Al foil and placed in an alumina boat in a box furnace for air calcination (Carbolite Gero Ltd.). The furnace was heated to 600 °C at 5 °C/min ramping rate and held for 1 h. ZrO_2_-based fibers were calcined at 800 °C for 1 h.

### Characterization

The viscosity of the spinning solutions at ambient conditions (temperature of 20.0 ± 1 °C) was measured by a Brookfield DV-II Viscometer with an HA torque spring and cone spindle CP-41. A fixed amount of 2 mL of solution was loaded into the sample cup. A ramping of rotation speed was used in 6 steps from 100 to 200 rpm, corresponding to 200 to 400 s^–1^ shear rate. The ramping was conducted twice, and the average viscosity values were reported. Scanning electron microscope (SEM) images were taken by a Zeiss Merlin, where the fiber samples were coated with 8 nm-Cr using a Leica ACE 600 Coater. The elemental line scans were obtained by a Zeiss Evo instrument for energy-dispersive X-ray spectroscopy (EDS) operated at 5 kV voltage. High-angle annular dark-field (HAADF) scanning transmission electron microscopy (STEM) images were acquired by a STEM detector equipped with a Zeiss Merlin. Transmission electron microscopy (TEM) images were obtained by a JEOL JEM-2100 at an operating voltage of 200 kV. The polymer fiber TEM and STEM samples were prepared by direct electrospinning a thin fiber layer onto 50 mesh copper grids (Agar Grids). Ceramic fiber samples for TEM and STEM observation were prepared by dispersing calcined ceramic fibers in ethanol and then drop casting the ethanol suspension onto a 300 mesh copper TEM grid followed by vacuum drying.

Fourier transform infrared (FTIR) attenuated total reflection (ATR) spectrum was recorded at 600 to 4000 cm^–1^ on a Shimadzu Fourier Transform Infrared Spectrophotometer IRSpirit. Thermal gravimetry analysis (TGA) was employed by using a PerkinElmer TGA 8000. The precursor samples were heated in air from room temperature to 900 °C at a rate of 10 °C/min. Nitrogen adsorption–desorption isotherms were recorded on a Micromeritics ASAP 2420 M system. The surface area and pore size distribution were determined by using Brunauer–Emmett–Teller (BET) and Barrett–Joyner–Halenda (BJH) methods. X-ray diffraction (XRD) was carried out on a Bruker D8 ADVANCE Eco X-ray diffractometer using Cu Kα radiation (wavelength λ = 0.154 nm). Lorentz functions were used to fit the crystalline peaks. The full width at half-maxima (fwhm’s) of the peaks were extracted from the fitting. The crystallite sizes (*D*) were obtained using the Scherrer equation *D* = *K*λ/(fwhm × cos θ), where the shape factor *K* is 0.9.

The tensile strengths of the samples were measured by a DMA TA Q800 machine in a single-cantilever configuration at room temperature. The fiber mats with thicknesses between 0.1 and 0.2 mm were cut into rectangular strips. These materials were clamped to produce a gauge length of 10 mm and tested at a tensile force ramping rate of 0.05 N/min (Figure 4m). The densities of the fiber materials were calculated by using the weight of the solid contents divided by the bulk volume of the test sample. The length, width, and thickness of the samples were measured by a digital caliper.

### Simulation of Thermomechanical Deformation

3D fiber models for finite element analysis were developed using COMSOL Multiphysics 6.1. Three core–shell fiber models, S25, S400, and S500, were built to model thermomechanical deformation behaviors at different temperature ranges. Based on the SEM/EDS results of the precursor fiber, S25 was designed as a straight core–shell fiber with a shell diameter of 1020 nm, a core diameter of 480 nm, and a length defined as 10 μm. S25 contains 11 fan-shaped cuts on the shell that are helically symmetrical about the fiber axis. All the cuts are 20 nm in width, spanned 90°, and the adjacent cuts are separated by 833 nm, with 90° difference in between. By increasing the temperature from 25 to 400 °C, S25 coiled into a helix with an average diameter of 762 nm. This deformed mesh was converted into the S400 model by removing the intersecting faces and edges, which was further used to simulate thermal deformation from 400 to 500 °C. Similarly, S500 was built based on the deformed S400, but modeled as a single-component structure because PVP is close to complete decomposition at 500–600 °C.

The thermomechanical deformation of single fibers was simulated using the Structural Mechanics and Heat Transfer in Solids module coupled with Thermal Expansion Multiphysics. Linear elastic materials were designed (with geometry nonlinearity included). Details are given in Table S1 (Supporting Information). At <400 °C, the Young’s modulus and Poisson’s ratio values are 10 GPa and 0.15 for the core component, close to a TiO_2_ xerogel,^80^ and 0.8 GPa and 0.32 for the shell, referring to typical PVP-based soft materials.^81,82^ Negative CTE values were used for all materials and varied depending on the temperature ranges. The CTE values were decided by ensuring that the dimensions of the deformed models match the SEM observations. Note that changing these values in a reasonable range (e.g., within 1 order of magnitude) would still lead to the formation of spring-shaped fiber morphology. To simulate the thermomechanical deformation of fiber models, a continuity restraint was set at the boundary pair of core and shell materials, and the fiber core was subjected to Rigid Motion Suppression in the Silid Mechanics module. Physics-controlled finer meshes were created, and a mesh convergence study was carried out. Stationary study was performed at each set temperature because the thermomechanical deformation herein does not depend on the time. Unless otherwise stated, the deformed fiber models were presented at a scale factor of 1.

In extended simulation studies, 20 μm-long straight core–shell fibers (S25-Extended) were built with material properties similar to those of S25. The cracks in the fiber shell have various geometrical symmetries and periodic lengths. The thermomechanical deformations of S25-Extended models were simulated from 25 to 400 °C. Moreover, to simulate the calcination of the supported precursor fibers, various boundary loadings were applied to both ends of the fiber model, which are tension stresses along the fiber axis and normal to the surface.

## Supporting Information Available

Figures S1 to S25: Photo and schematic of two solutions feeding through a coaxial nozzle; TEM images of core–shell precursor fiber; SEM images and FTIR spectra of washed precursor fibers; SEM of TiO_2_ fibers in different morphologies, TiO_2_ fibers from varied core feeding rates, TiO_2_ fibers from blended solutions; pore size distribution of TiO_2_ fibers; contraction percentages of fiber diameter before and after calcination; ATR-FTIR spectra of various precursor fibers and pure PVP fiber; plot of the geometrical parameters of springs; ATR-FTIR spectra and SEM images of T/P fibers upon heating; STEM images of the core–shell precursor fibers; SEM images of coelectrospun TiO_2_ fibers at varied feeding rates; COMSOL simulation of the thermomechanical deformation of the core–shell fibers in various geometries; the solution viscosities, crystallite sizes, and SEM images of various ceramic fibers; SEM and EDS spectra of ZrO_2_/Al_2_O_3_ fibers; SEM images of the TiO_2_/SiO_2_ springs; raw data of the strain–stress curves of ceramic fibers; Tables S1 and S2: information on the 3D fiber models for COMSOL Multiphysics simulation; composition of the spinning solutions used to prepare TiO_2_ fibers (PDF)
Digital movie of the sol/polymer co-electrospinning process (AVI)
Digital movie of electrospinning a nonspinnable sol (AVI)
Deformation and recovery of flexible T@P fibers (AVI)
Fracture of brittle T/P fibers (AVI)

## Supplementary Material

nn3c12659_si_001.pdf

nn3c12659_si_002.avi

nn3c12659_si_003.avi

nn3c12659_si_004.avi

nn3c12659_si_005.avi
