# Supplementary material for: Electrospinning Nonspinnable Sols to Ceramic Fibers and Springs
Source: ACS Nano. 2024 May 8;18(21):13538–50. doi: 10.1021/acsnano.3c12659 (PMC11140837; doi:10.1021/acsnano.3c12659)
Supplement: Supplementary file 1 — nn3c12659_si_001.pdf [file nn3c12659_si_001.pdf]

Electrospinning Non-Spinnable Sols to Ceramic Fibers and Springs

Shiling Dong [1], Barbara M. Maciejewska [1], Ryan M. Schofield [1], Nicholas Hawkins [2], Clive R. Siviour [2], Nicole Grobert [1*]*

1 Department of Materials, University of Oxford; Parks Road, Oxford OX1 3PH, UK

2 Department of Engineering, University of Oxford, Parks Road, Oxford OX1 3PJ, UK

* Email: barbara.maciejewska@materials.ox.ac.uk; nicole.grobert@materials.ox.ac.uk

This PDF file includes:

Figure S1 to S25……………………………………………………….………...……...… 2

Table S1 and S2…………………………….…………………………….………...…….… 27

Captions for Movies S1-S4………………..……….…………………..……...…….……… 29

Reference…………………………………………………………………...……...….…… 29

Other Supplementary Materials for this manuscript include the following:

Movies S1-S4

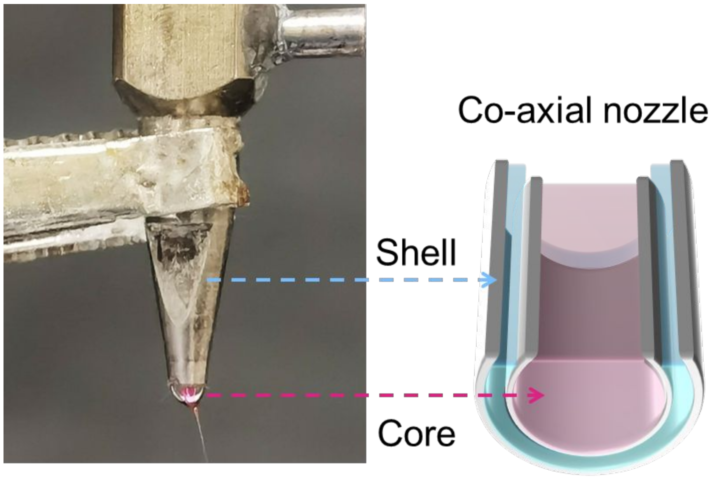

Figure S1. Digital photo and schematic showing the two solutions feeding through the co-axial nozzle as the core and the shell.

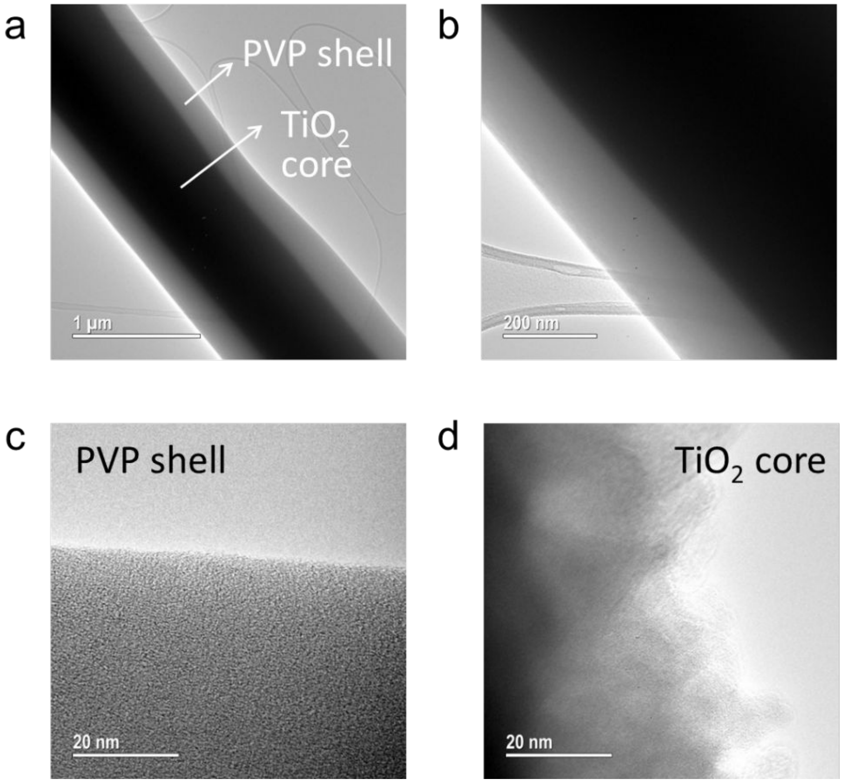

Figure S2. (a) TEM image of the core-shell TiO_2@PVP fibers, and (b) close-ups of the core-shell boundary. (c) TEM image revealing the amorphous state of the PVP shell, and (d) the TiO_2 core possessing a typical ceramic xerogel microstructure. [1]

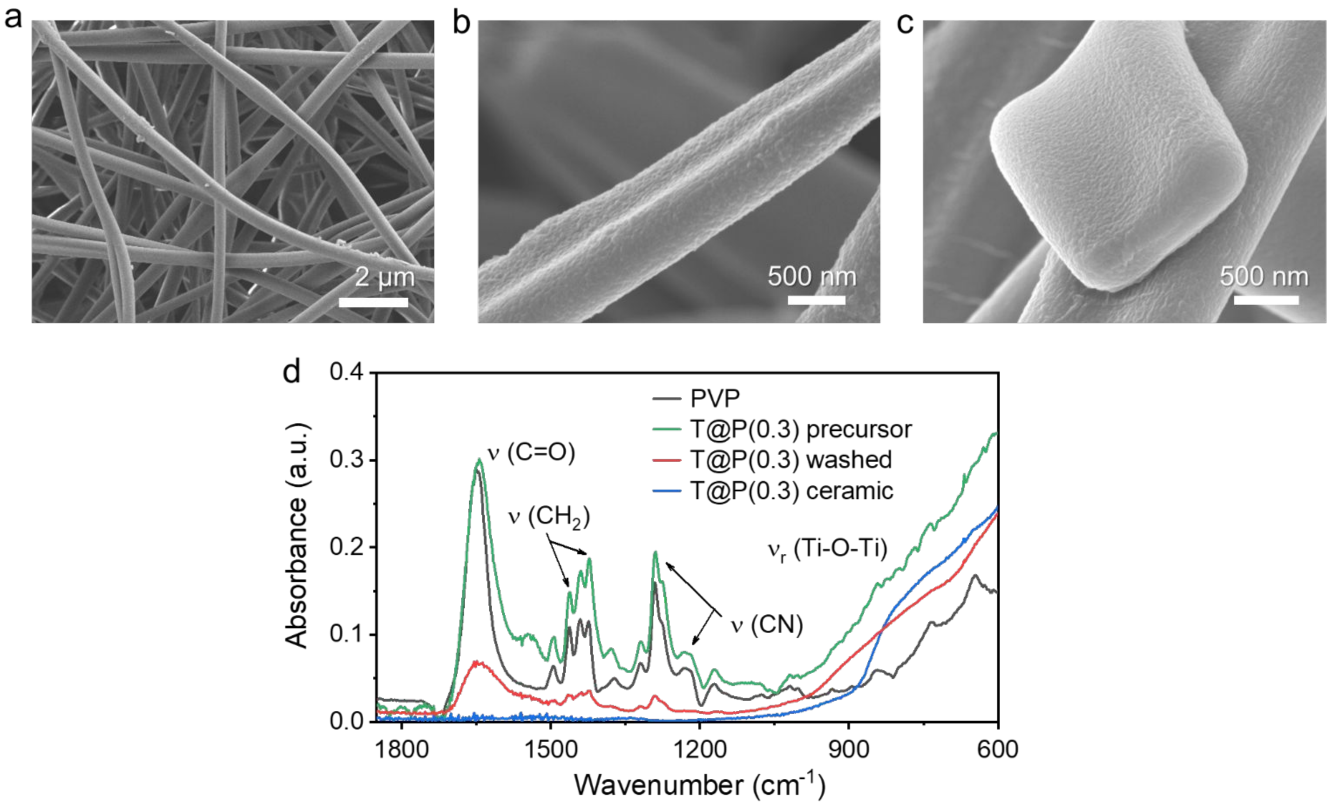

Figure S3. (a-c) SEM images of core-shell TiO_2@PVP fibers (T@P(0.3)) after soaking in water for several days to dissolve the PVP shell. (d) Attenuated total reflection-Fourier transform infrared spectrum (ATR-FTIR) of T@P(0.3) fiber at different synthesis stages. The washed fiber shows significantly weakened C-N absorption peaks, indicating the removal of the PVP shell.

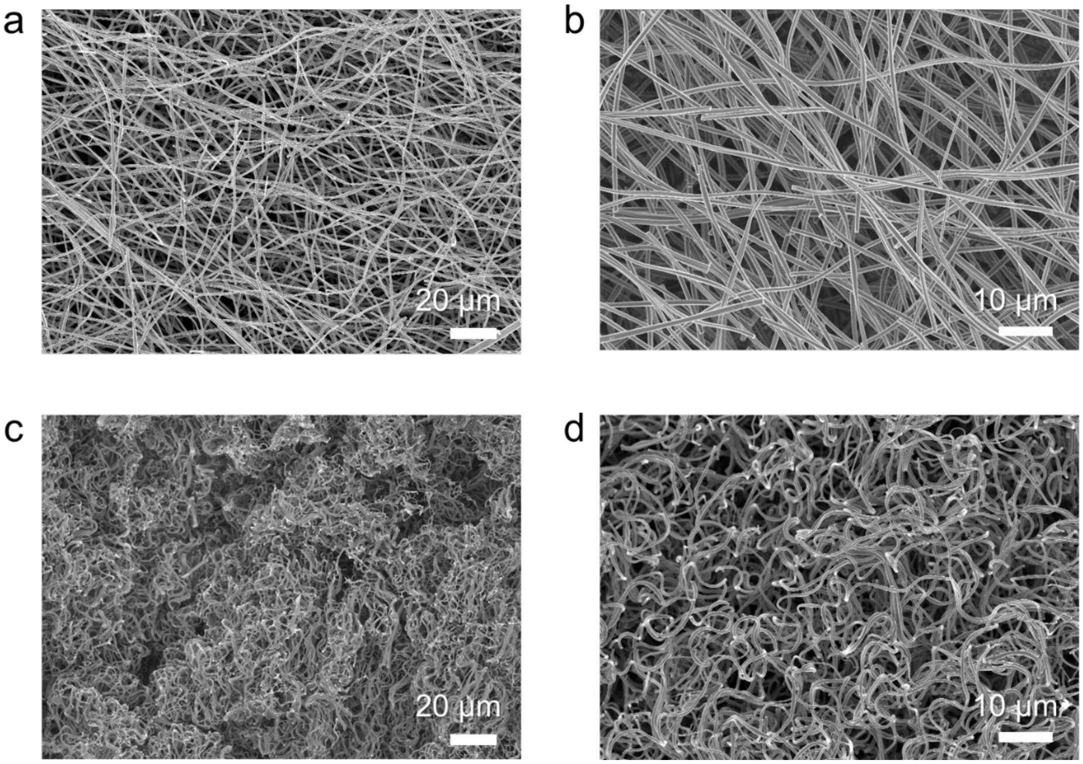

Figure S4. Low-magnification SEM images of TiO_2 fibers in (a, b) straight fiber morphology after calcination supported by Al foil substrate, and (c, d) coiled nanospring morphology if the substrate is removed before calcination.

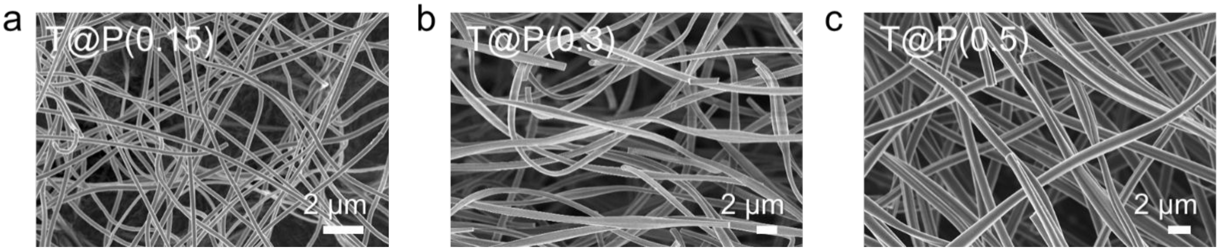

Figure S5. SEM images of TiO_2 fibers obtained from TiP sol/PVP solution co-electrospinning where the shell solution is fed at 1 mL/h while the core sol is fed at (a) 0.15 mL/h, (b) 0.3 mL/h, and (c) 0.5 mL/h.

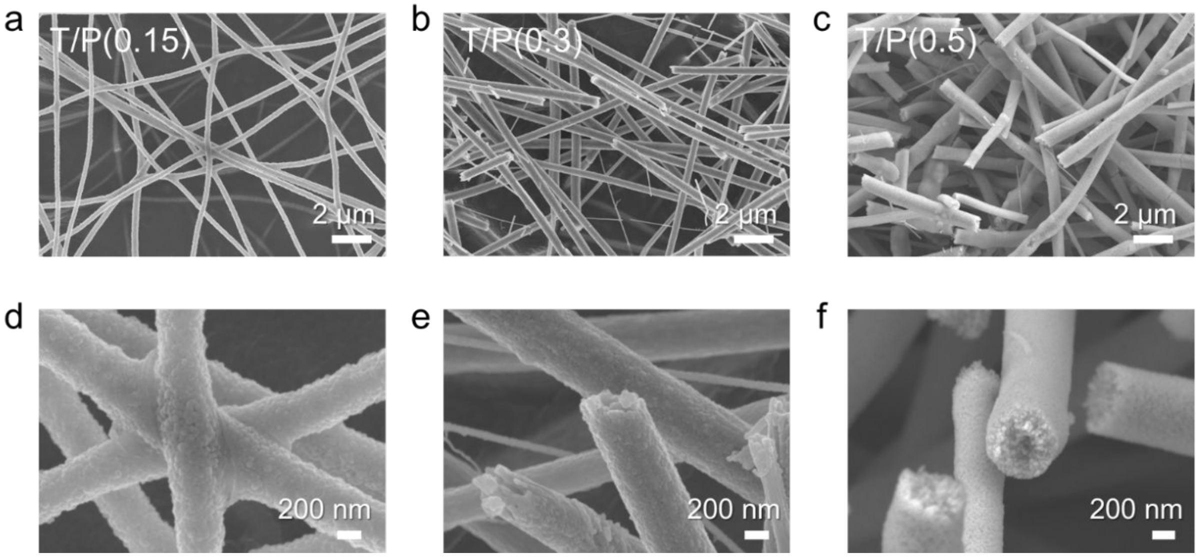

Figure S6. SEM images of TiO_2 fibers obtained from TiP sol/PVP solution mixed at varied ratios and electrospun at designated flow rates, (a, d) 0.15: 1 ratio and 1.15 mL/h, (b, e) 0.3: 1 ratio and 1.3 mL/h, and (c, f) 0.5: 1 ratio and 1.5 mL/h.

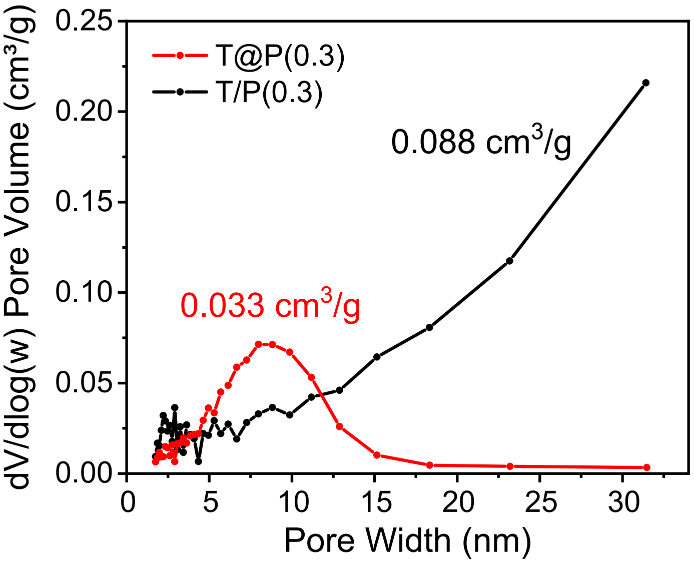

Figure S7. Barrett-Joyner-Halenda (BJH) mesopore size distribution curves of T@P(0.3) and T/P(0.3) fibers. The calculated total pore volumes are labeled.

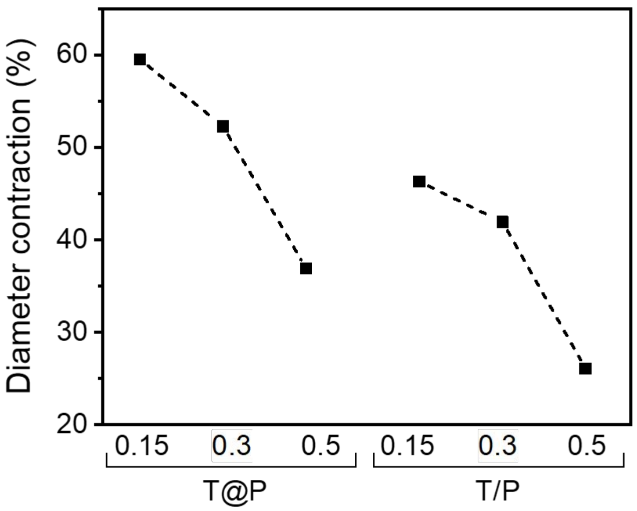

Figure S8. The reduced percentages of the fiber diameter before and after calcination.

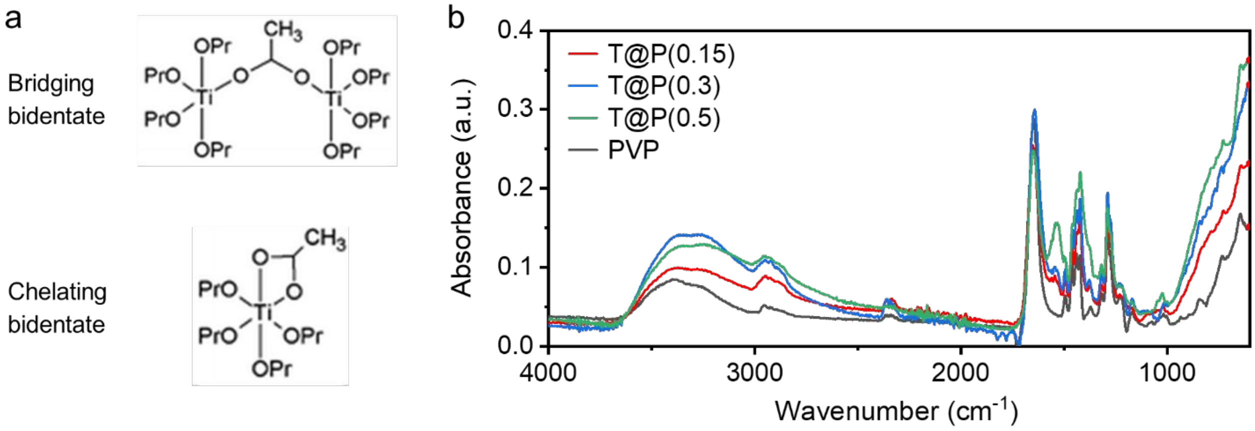

Figure S9. (a) Schematics of Ti-acetate complex in bridging bidentate and chelating bidentate forms. [2] (b) ATR-FTIR spectra of T@P precursor fibers and pure PVP fiber.

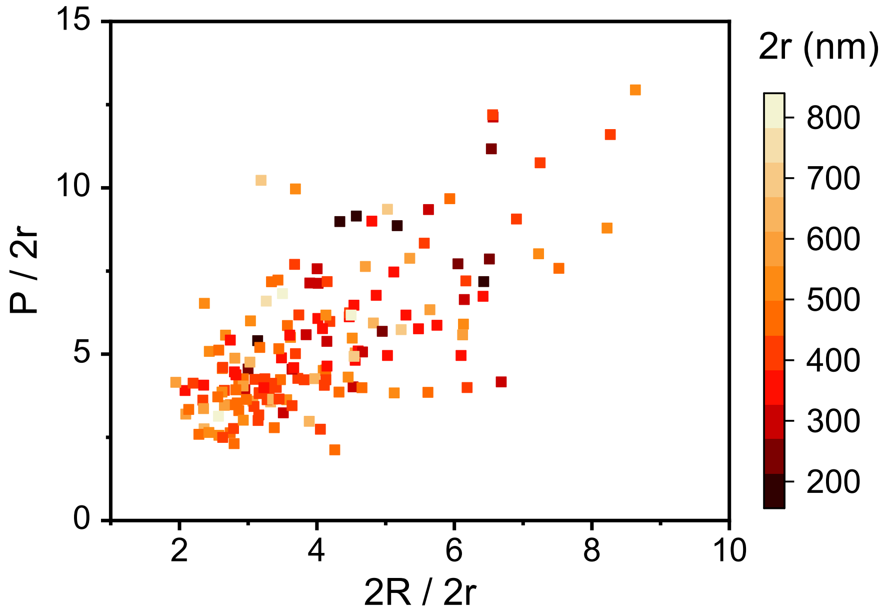

Figure S10. Scatter plot of the P/2r against 2R/2r of each ceramic spring. The color map indicates the fiber diameter (2r).

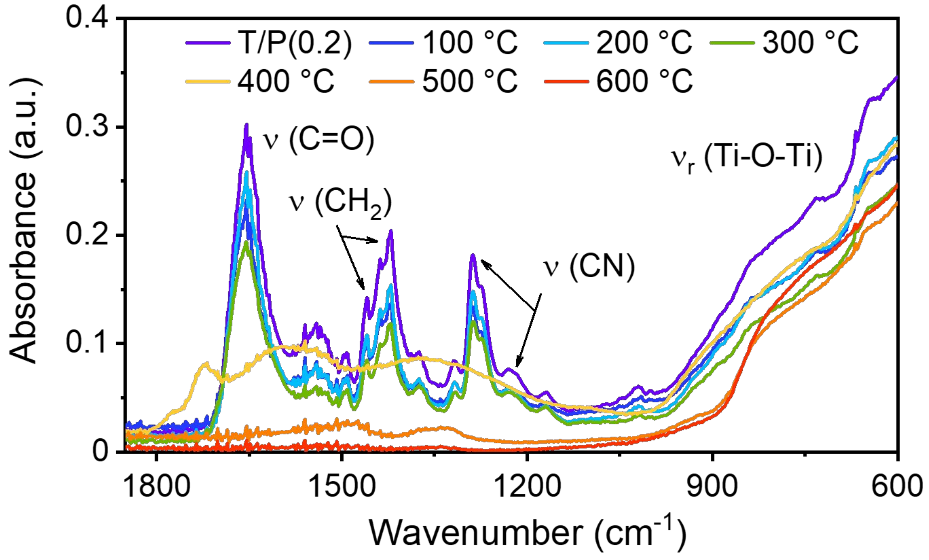

Figure S11. ATR-FTIR spectra of T/P(0.2) precursor fibers at room temperature and upon heat treatment at 100-600 °C.

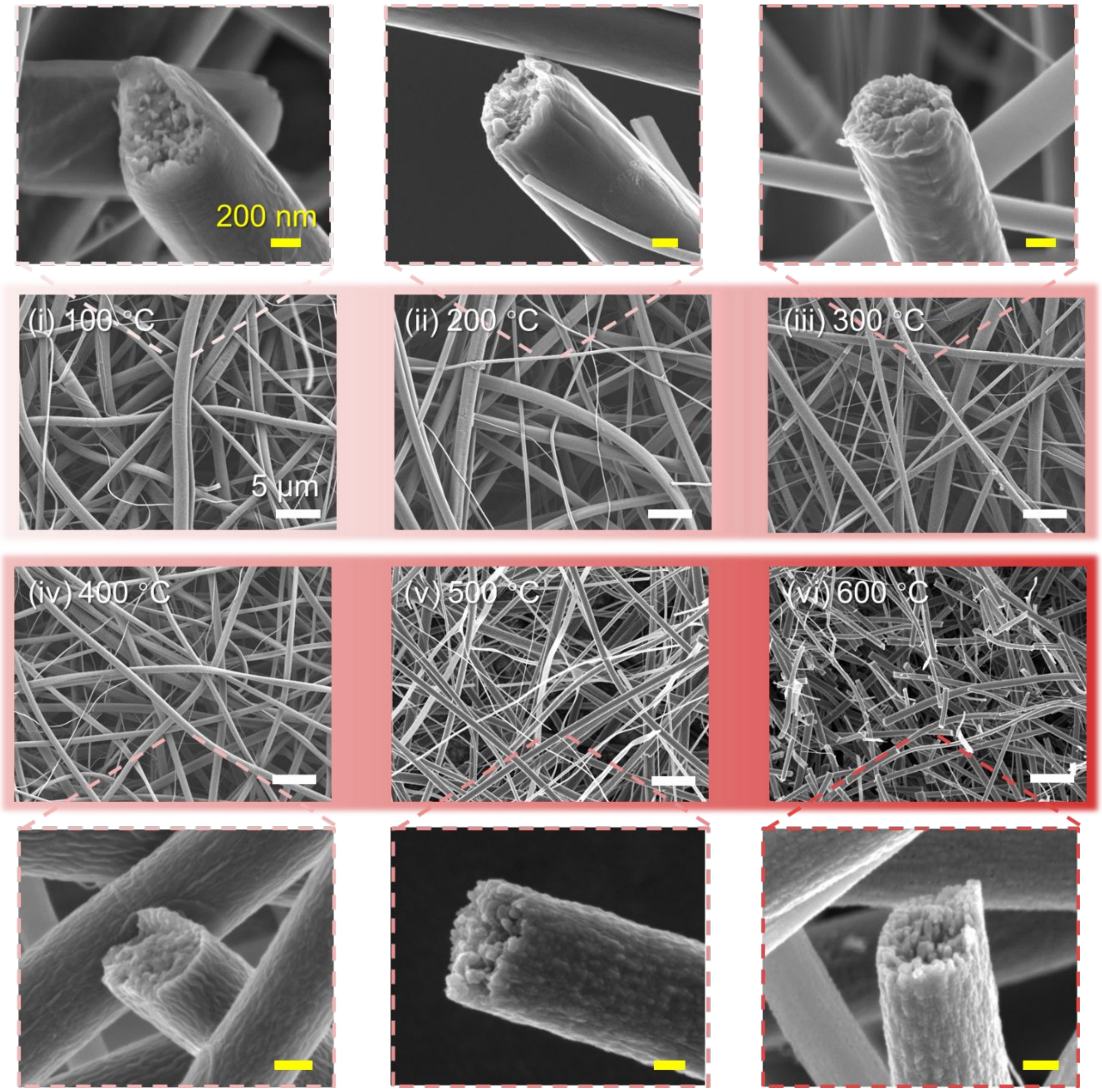

Figure S12. SEM images of T/P (0.2) precursor fibers show the structural evolution upon increasing the temperature to 100-600 °C. The white scale bars for low-magnification images are 2 μm. The yellow scale bars for high-magnification images represent 200 nm.

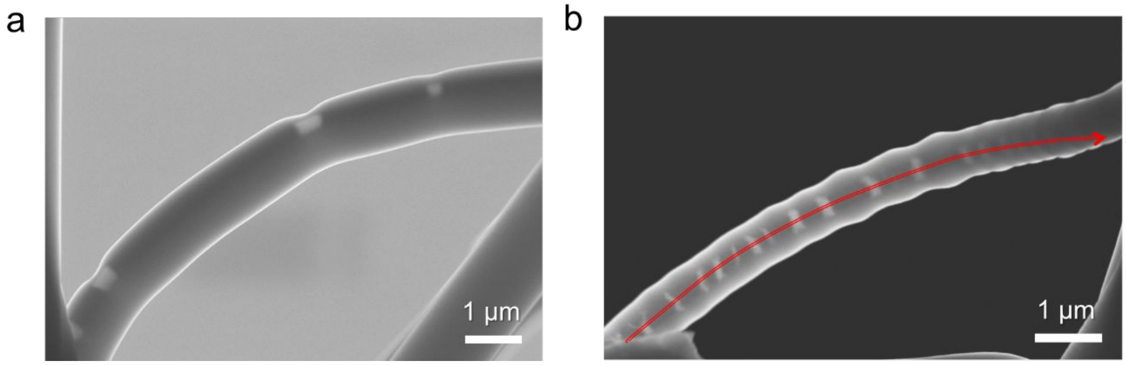

Figure S13. Dark-field STEM images of the core-shell T@P precursor fibers display (a) the cracks distributed at the outer side of the curved fibers and (b) the rotation of cracks upon changing the curving direction.

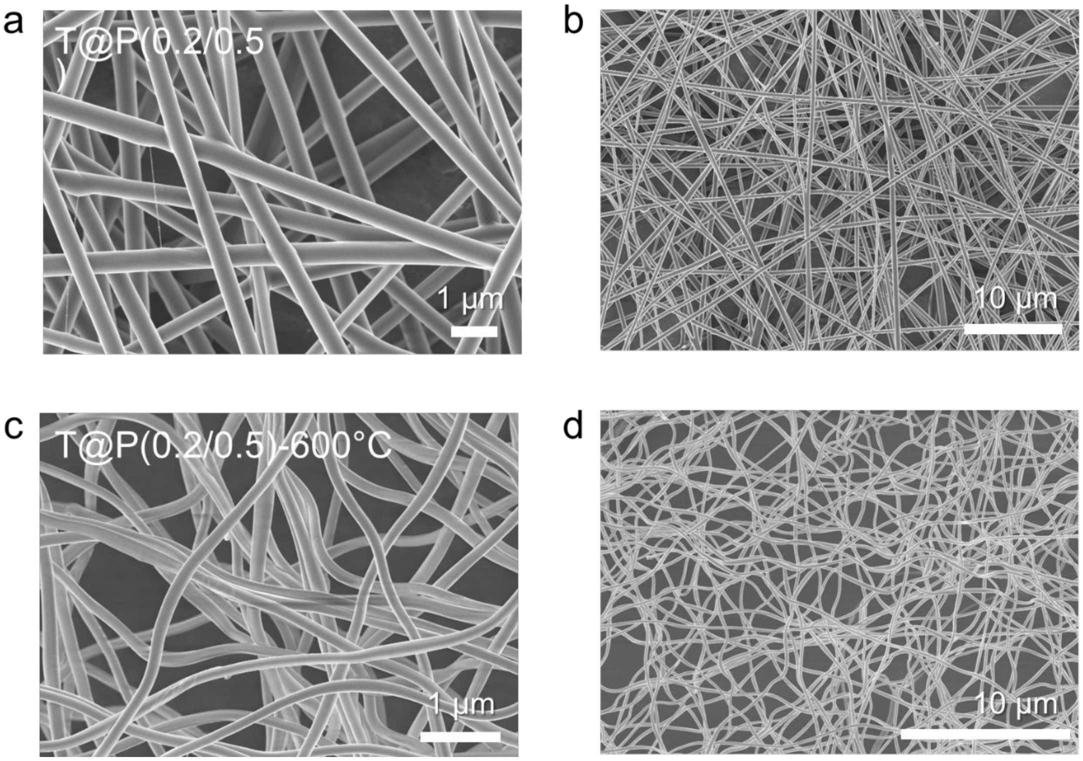

Figure S14. SEM images of fibers electrospun at TiP sol fed at 0.2 mL/h and PVP solution fed at 0.5 mL/h, respectively. (a, b) As-electrospun core-shell fibers and (c, d) after calcination.

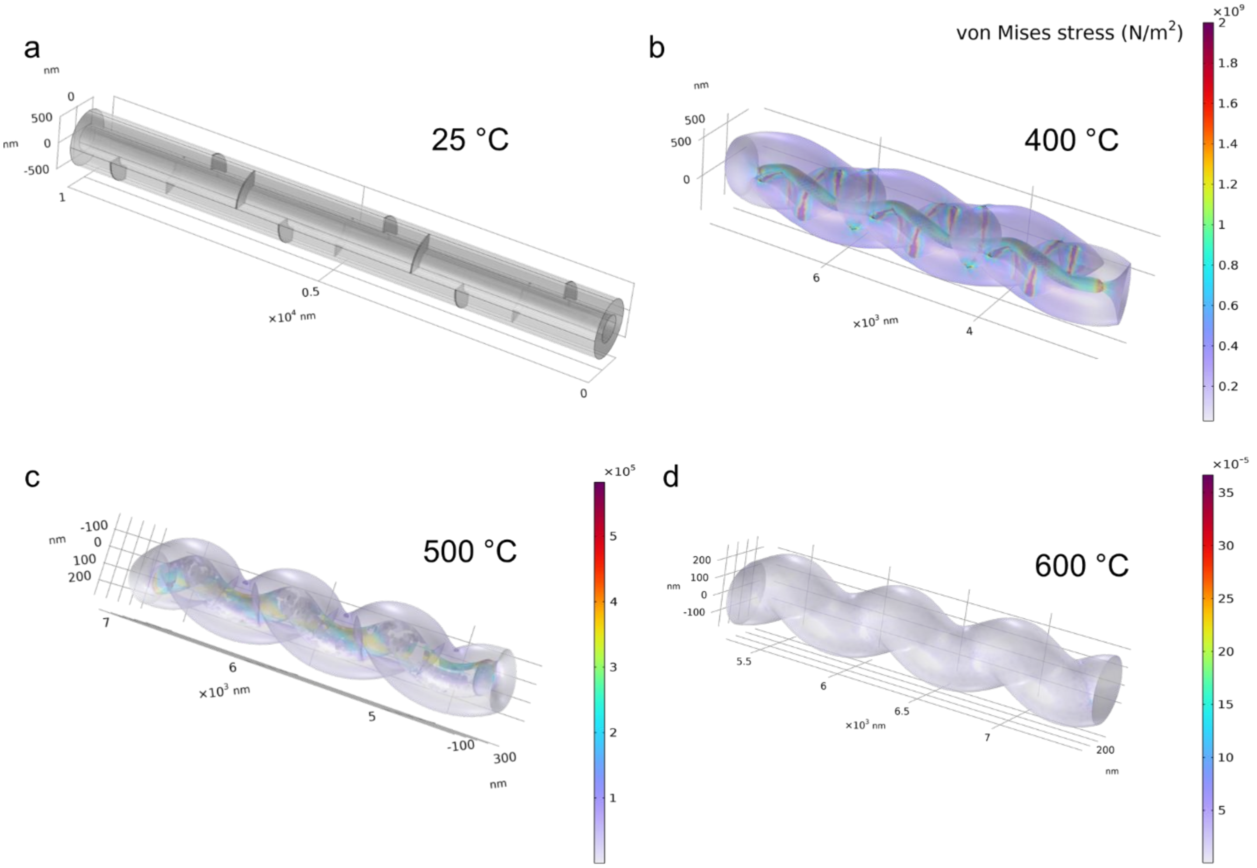

Figure S15. COMSOL simulation of the thermo-mechanical deformation of the 3D core-shell fibers, showing the original model (a) and the deformed structure at 400 °C (b), 500 °C (c), and 600 °C (d).

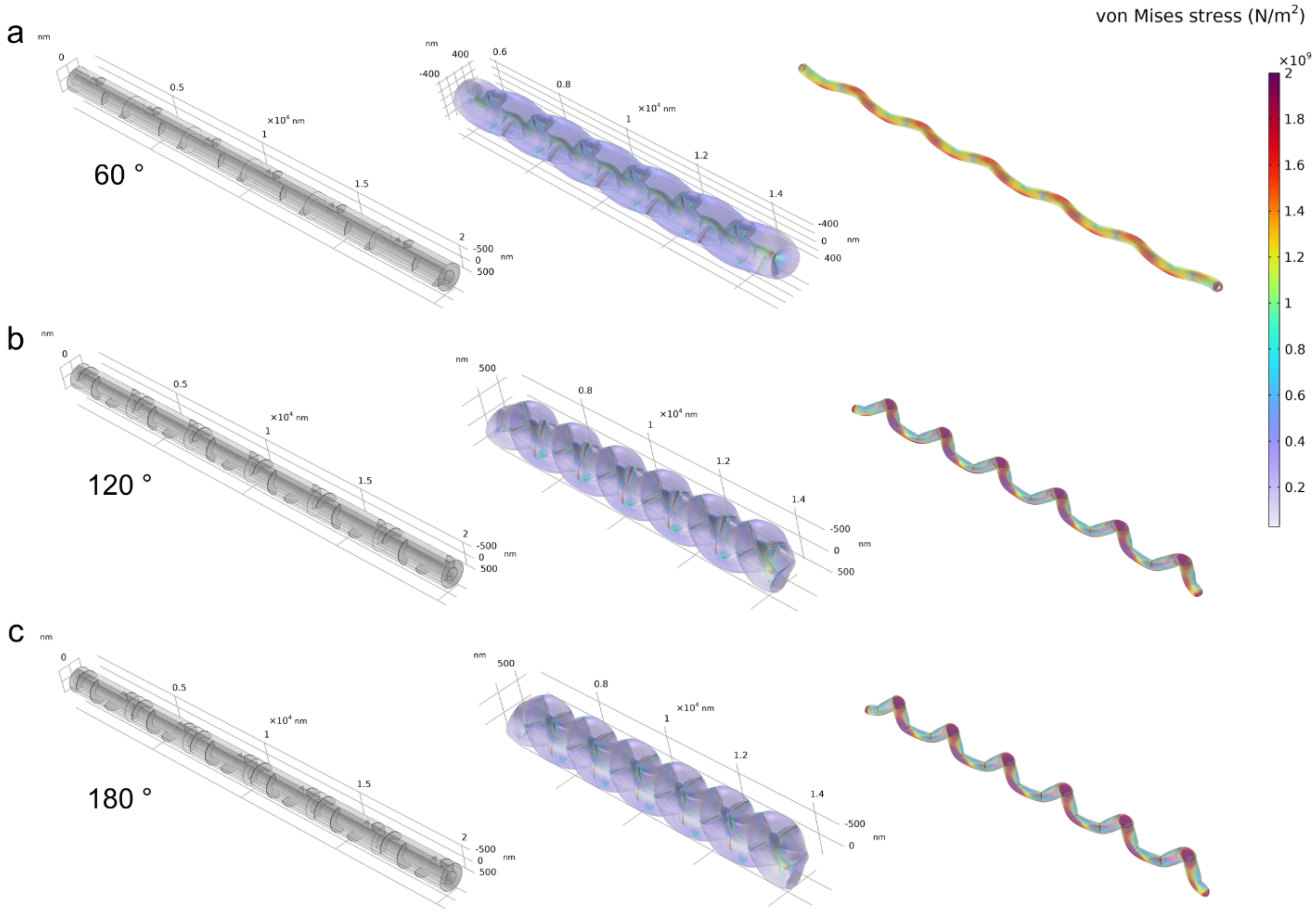

Figure S16. COMSOL simulation of the thermal contraction of the 3D core-shell fiber with fan-shaped cracks spanned (a) 60 °, (b) 120 °, and (c) 180 °. The deformed models are shown as the core-shell fibers and the core part only (scale factor of 1), following the same color scale representing the von Mises stress.

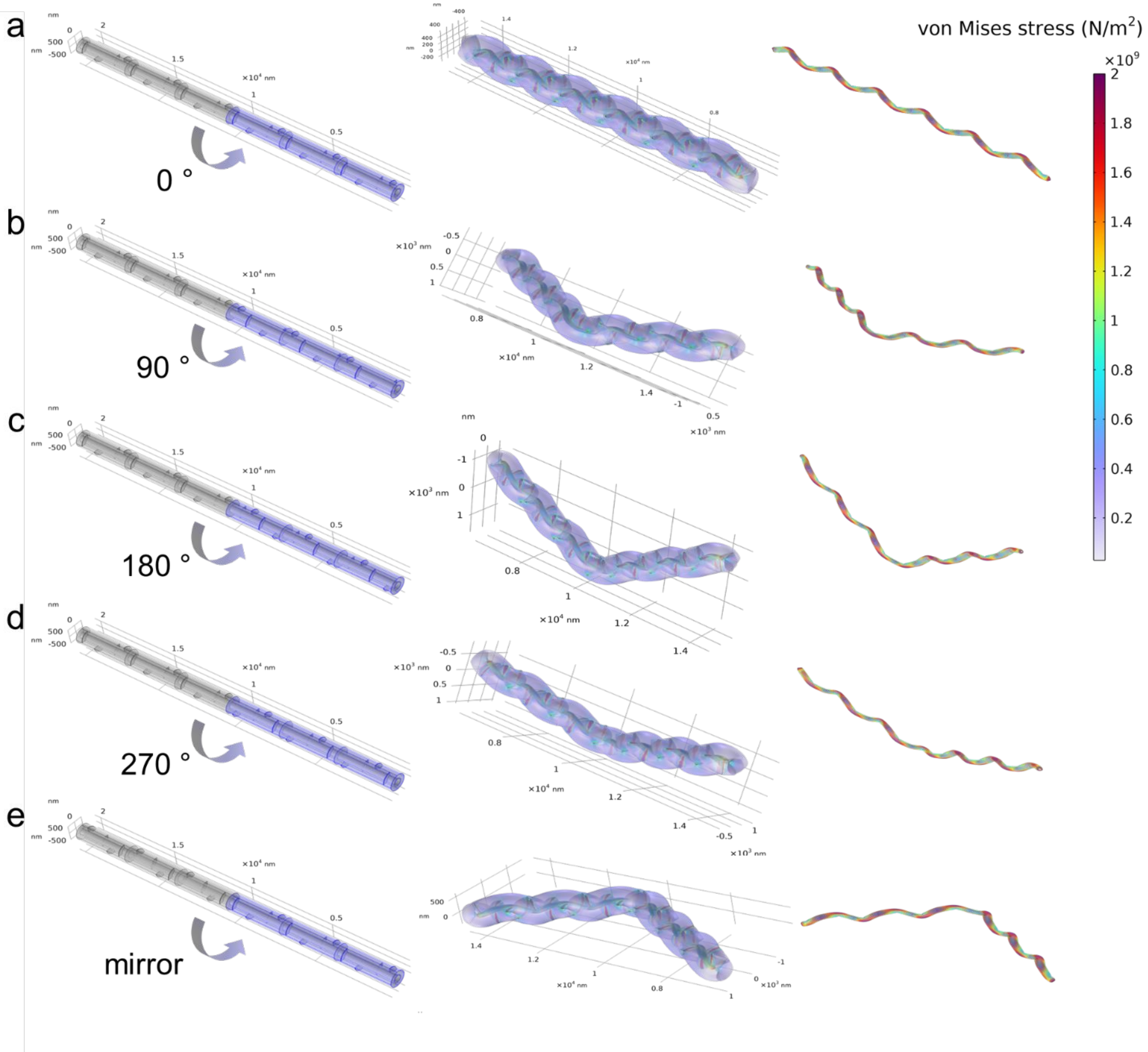

Figure S17. COMSOL simulation of the thermal contraction of the 3D core-shell fiber with half of the fiber structure rotated by different degrees (a-d) or mirrored (e). The deformed models are shown at a scale factor of 0.75, following the same color scale representing the von Mises stress.

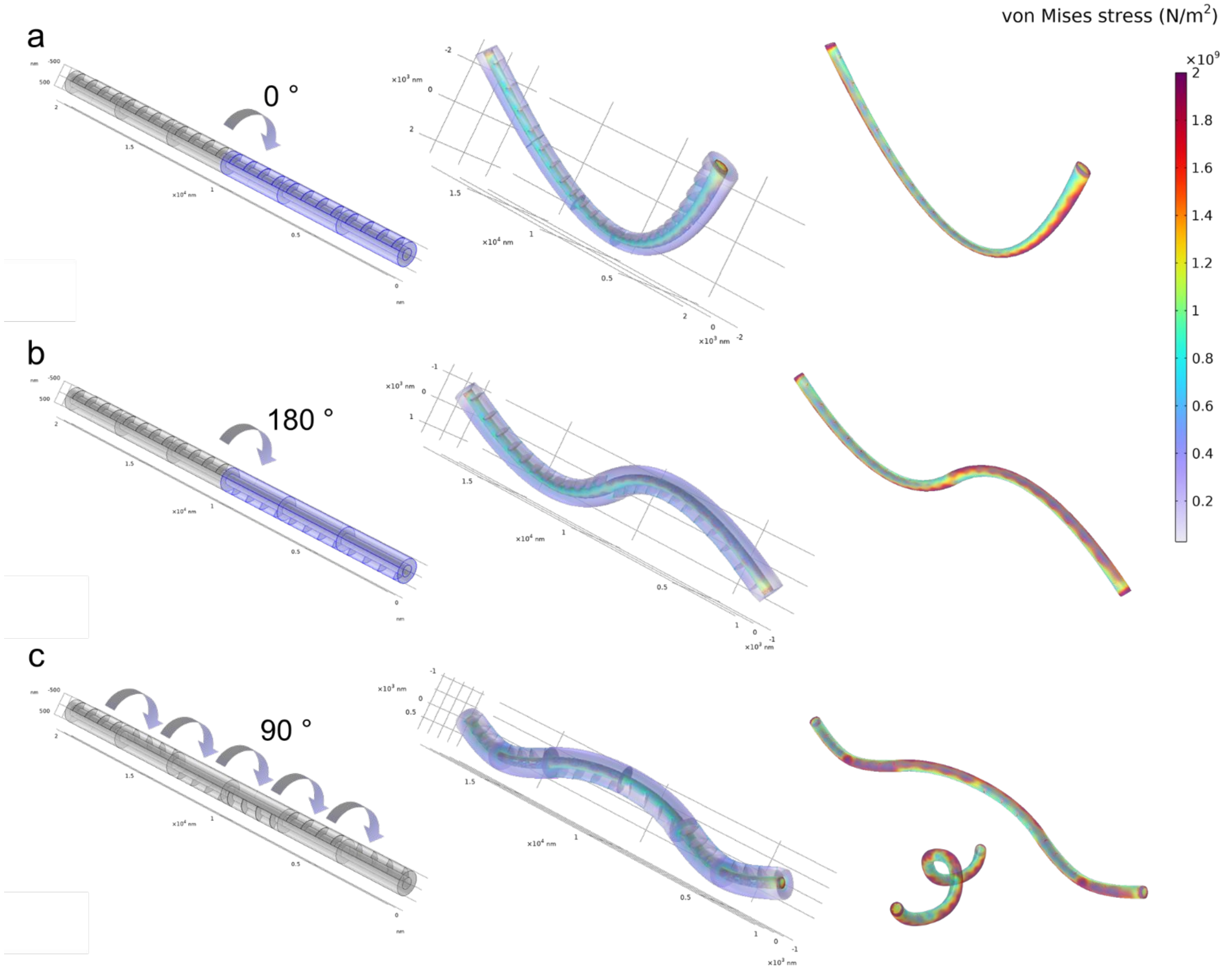

Figure S18. COMSOL simulation of the thermal contraction of the 3D core-shell fiber with cracks aligned on one side of the shell with different periodicities. The deformed models are shown at a scale factor of 0.4, following the same color scale representing the von Mises stress.

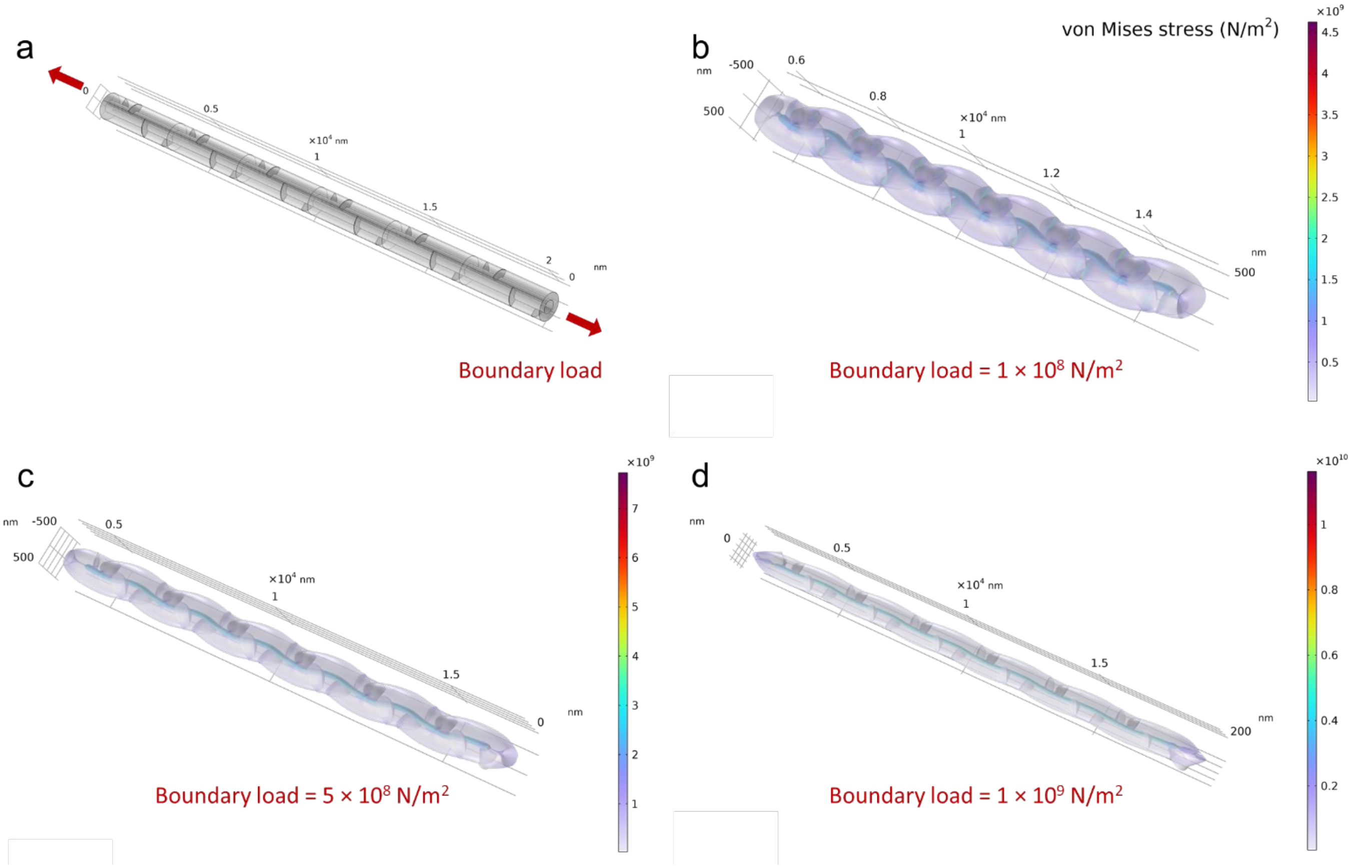

Figure S19. COMSOL simulation of the thermal contraction of the 3D core-shell fiber with cracks aligned on one side of the shell with different periodicities. The scale factor is 1.

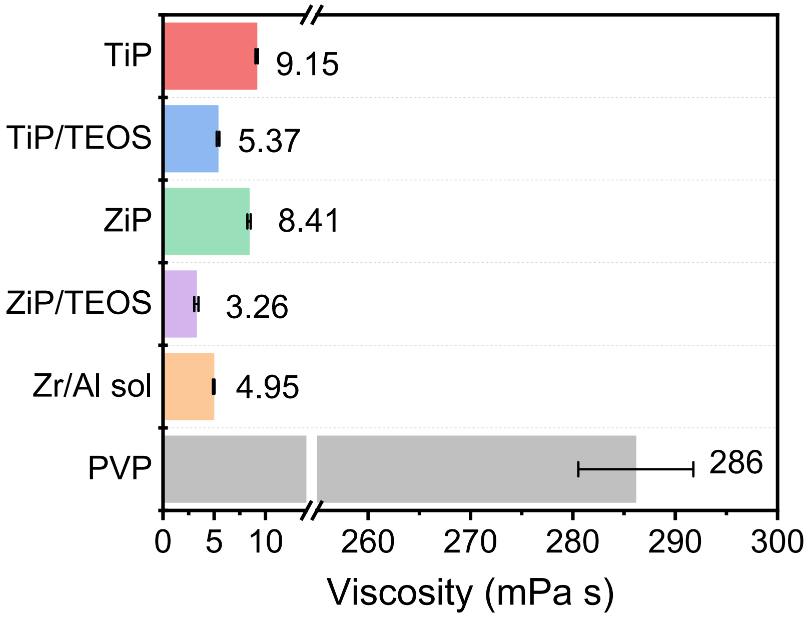

Figure S20. The viscosities of the dilute sol used in the sol/polymer co-electrospinning compared to the PVP solution.

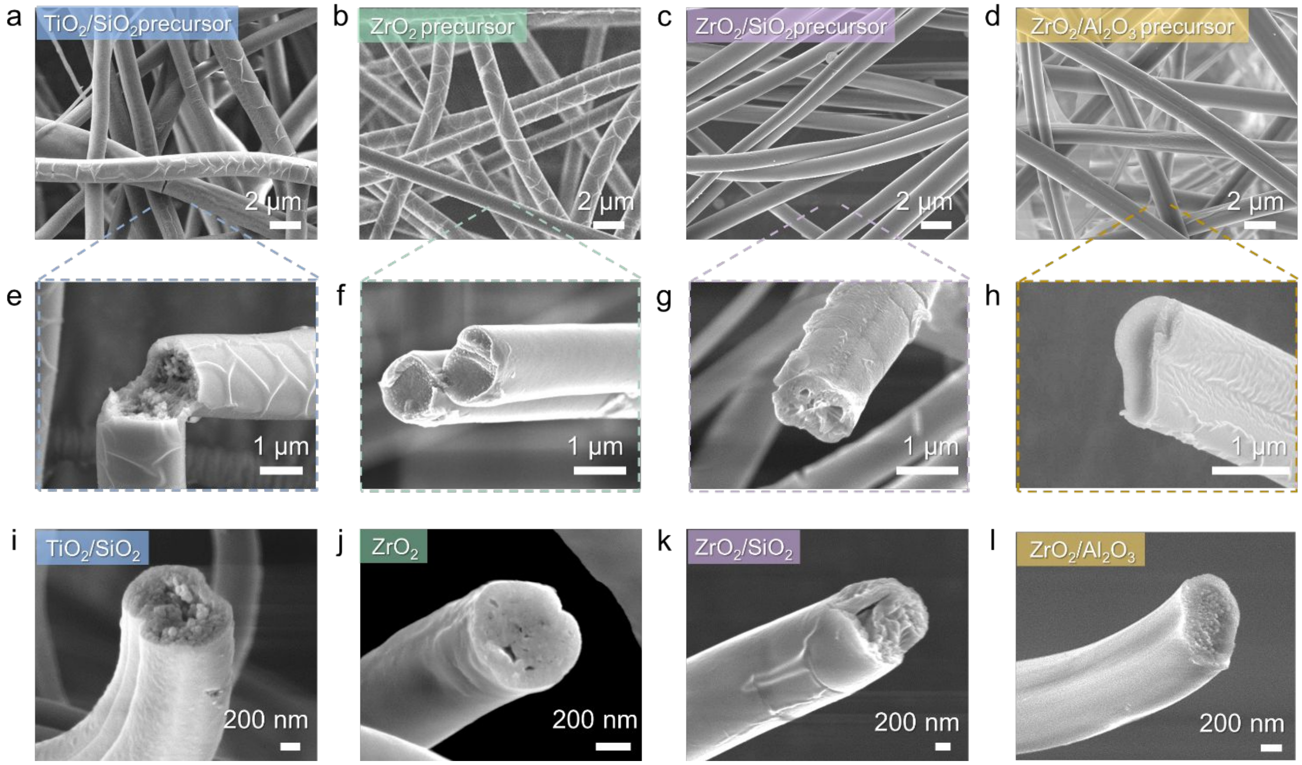

Figure S21. SEM images of the core-shell precursor fiber of (a, e) TiO_2/SiO_2, (b, f) ZrO_2, (c, g) ZrO_2/SiO_2, and (d, h) ZrO_2/Al_2O_3. SEM images of the calcined ceramic fiber made of (i) TiO_2/SiO_2, (j) ZrO_2, (k) ZrO_2/SiO_2, and (l) ZrO_2/Al_2O_3.

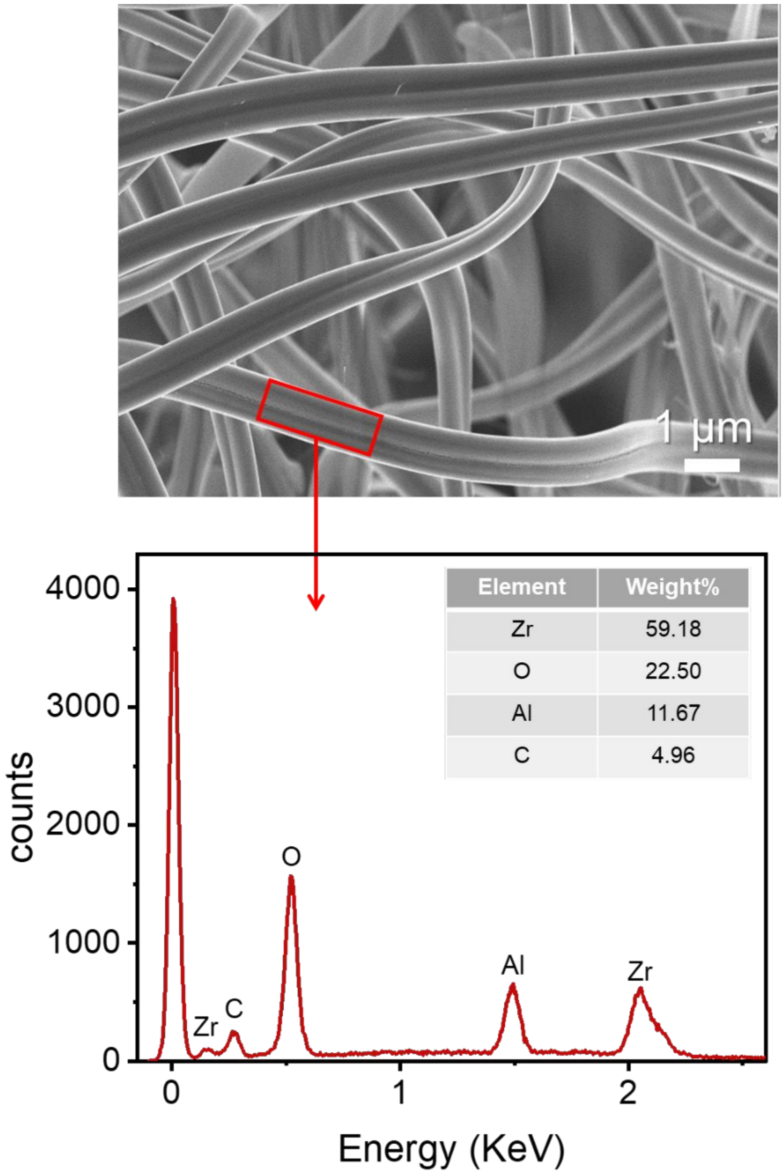

Figure S22. SEM image and EDS spectra of the ZrO_2/Al_2O_3 ceramic fiber produced by co-electrospinning dilute aqueous Al/Zr sol.

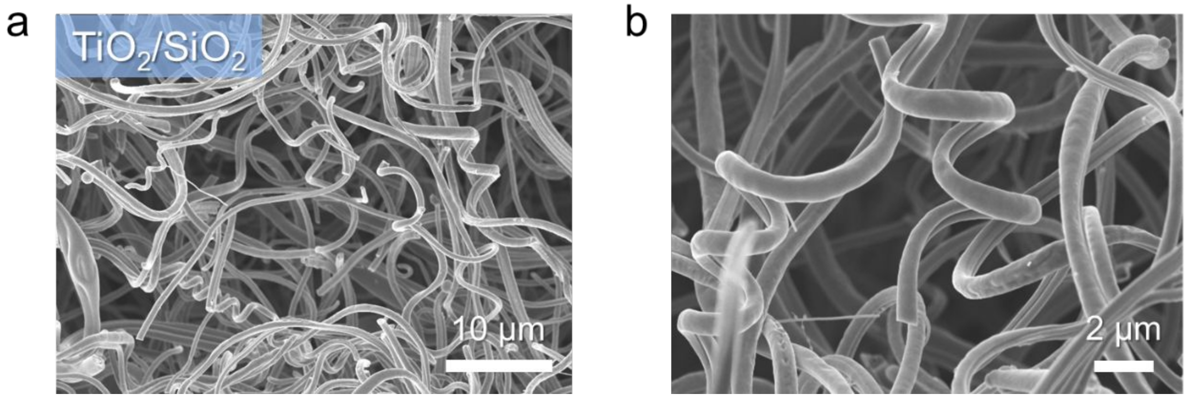

Figure S23. SEM images of TiO_2/SiO_2 ceramic springs.

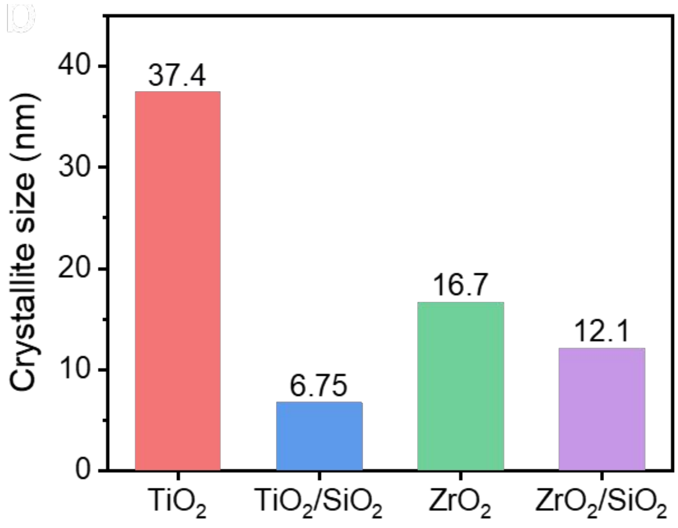

Figure S24. Crystallite sizes of various ceramic fibers calculated based on XRD spectra using the Scherrer equation.

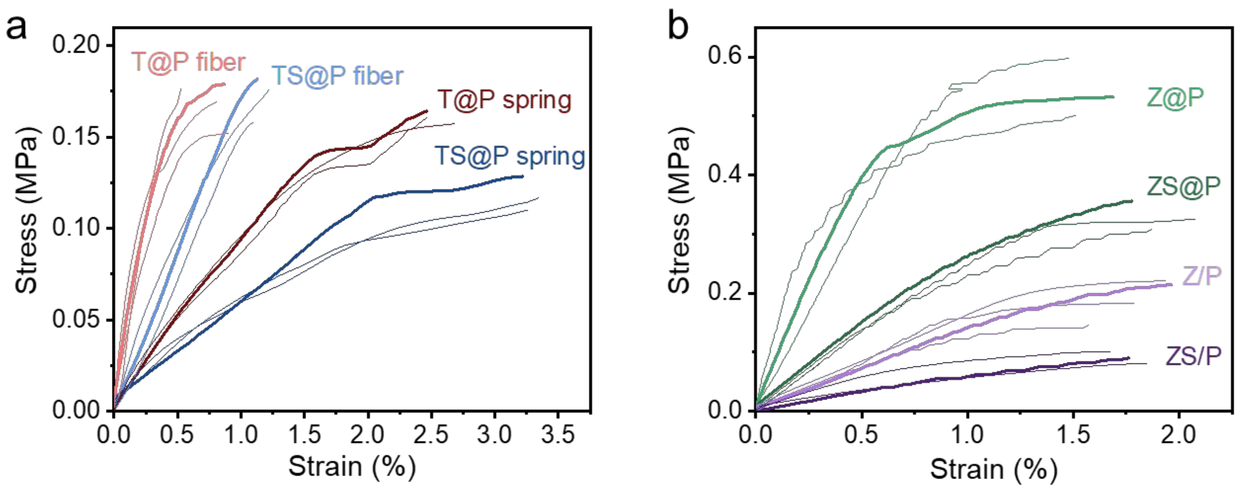

Figure S25. (a) Tensile stress-strain curves of TiO_2 (T@P) and TiO_2/SiO_2 (TS@P) straight fibers and springs. Multiple tests have been conducted on each type of fiber sample to ensure the measured Young's modulus and toughness values are within reasonable error ranges. Some of these results are shown. The bold lines represent the results we report in the manuscript. (b) As-measured tensile stress-strain curves of ZrO_2 and ZrO_2/SiO_2 fibers created by co-electrospinning (Z@P, ZS@P) and conventional sol-gel electrospinning (Z/P, ZS/P).

Table S1. The geometry information of the 3D fiber models, and the material properties used in the COMSOL Multiphysics simulation.

Model name	Fiber structure	Diameter	CTE	Young's modulus	Poisson's ratio	Simulation temperature range	Output fiber diameter
		[nm]	[10^{-4} 1/K]	[GPa]		[°C]	[nm]
S25	Shell	1023	{-1.8, -1.8, -0.4}	0.8	0.32	25 – 400	761.7
	Core	480	-19	10	0.15		
S400	Shell	746	{-52, -52, -6}	8E-5	0.1	400 – 500	372.3
	Core	353	-52	10	0.15		
S500	Uniform	374	{-6, -6, -1}	30	0.2	500 – 600	353.5
S25-Extended	Shell	1023	{-1.8, -1.8, -0.4}	0.8	0.32	25 – 400	
	Core	480	-19	10	0.15		

Table S2. Composition of the spinning solutions used to prepare TiO_2 fibers.

Sample name	Solutions	Mixed ratio [vol%/ vol%]	Flow rate [mL/h]
T@P(0.15)	Alkoxide sol: 4.3 g TiP and 1 g AcOH<br><br>Polymer solution: 1 g PVP and 10 ml EtOH	Separated	Core: 0.15<br>Shell: 1
T@P(0.2)		Separated	Core: 0.2<br>Shell: 1
T@P(0.3)		Separated	Core: 0.3<br>Shell: 1
T@P(0.5)		Separated	Core: 0.5<br>Shell: 1
T/P(0.15)		0.15: 1	1.15
T/P(0.2)		0.2: 1	1.2
T/P(0.3)		0.3: 1	1.3
T/P(0.5)		0.5: 1	1.5

Movie S1. The digital movie of sol/polymer co-electrospinning process where the core sol is dyed by Rhodamine B into a dark pink color.

Movie S2. The digital movie of electrospinning a non-spinnable sol. Upon applying the voltage, the fast drying of the reactive sol at the nozzle tip causes total nozzle blockage, and no fiber is electrospun.

Movie S3. The digital movie of the brittle T/P fiber mat produced by conventional sol-gel electrospinning.

Movie S4. The digital movie of the deformation and recovery of the flexible T@P fiber mat produced by sol/polymer co-electrospinning.

Reference

(1) Lee, E.-J.; Jun, S.-H.; Kim, H.-E.; Kim, H.-W.; Koh, Y.-H.; Jang, J.-H. Silica Xerogel-Chitosan Nano-Hybrids for Use as Drug Eluting Bone Replacement. *J. Mater. Sci. Mater.*

Med. **2010**, *21*, 207–214.

(2) Perrin, F. X.; Nguyen, V.; Vernet, J. L. FT-IR Spectroscopy of Acid-Modified Titanium Alkoxides: Investigations on the Nature of Carboxylate Coordination and Degree of Complexation. *J. Sol-Gel Sci. Technol.* **2003**, *28* (2), 205–215.
